# Eocene amber fossils reveal how complex trophic interactions shaped tropical rainforest biodiversity

**DOI:** 10.1016/j.isci.2025.113430

**Published:** 2025-08-25

**Authors:** Priya Agnihotri, Vikram Partap Singh, Hukam Singh, David Grimaldi, Mahesh G. Thakkar, Tanu Priya, K.A. Subramanian, Suryendu Dutta, Shreya Mishra

**Affiliations:** 1 Birbal Sahni Institute of Palaeosciences, 53-University Road, Lucknow 226 007, Uttar Pradesh, India; 2 American Museum of Natural History, 200 Central Park West, New York, NY 10024-5102, USA; 3 Department of Earth Sciences, Indian Institute of Technology Bombay, Mumbai 400 076, India; 4 Southern Regional Centre, Zoological Survey of India, Chennai 600 028, India

**Keywords:** Paleontology, Interaction of plants with organisms, Paleobiology, Interaction of plants with arthropods

## Abstract

The Eocene Epoch represented a pinnacle in Indian paleobiodiversity, explained by the ESAT (energy-stability-area-time) theory, which links climatic stability and geological time in fostering immense biodiversity. We provide a reconstruction of a middle Eocene tropical ecosystem from an amber biota recovered from the Harudi Formation (∼41.6 ± 0.5 to ∼40.8 ± 0.5 Ma), Umarsar Lignite Mine (ULM), western India. It reveals a highly diverse ecosystem (>800 arthropods of various taxonomic ranks along with 78 genera and 118 species of palynomorphs) thriving in warm and humid conditions (mean temperature ∼25°C; rainfall ∼2,450 mm/year), analogous to modern tropical climates. The findings show that favorable climate, ecological complexity, and India’s northward drift facilitated tropical lineage diversification, reinforcing ESAT as a robust explanatory model for deep-time biodiversity patterns. These findings also offer valuable analogs for predicting how biodiversity and functional networks in current tropical forests might respond to ongoing climate change, emphasizing the need to conserve both species and their ecological interactions.

## Highlights

• Reconstructs Eocene tropical rainforest ecosystems using fossilized amber biota
• Emphasizes climate stability, area, and time in diversity—supports ESAT theory
• Demonstrates ancient origins of modern tropical lineages in India
• Shows biodiversity resilience to past climate shifts—vital for future predictions

## Graphical abstract

**Figure:**
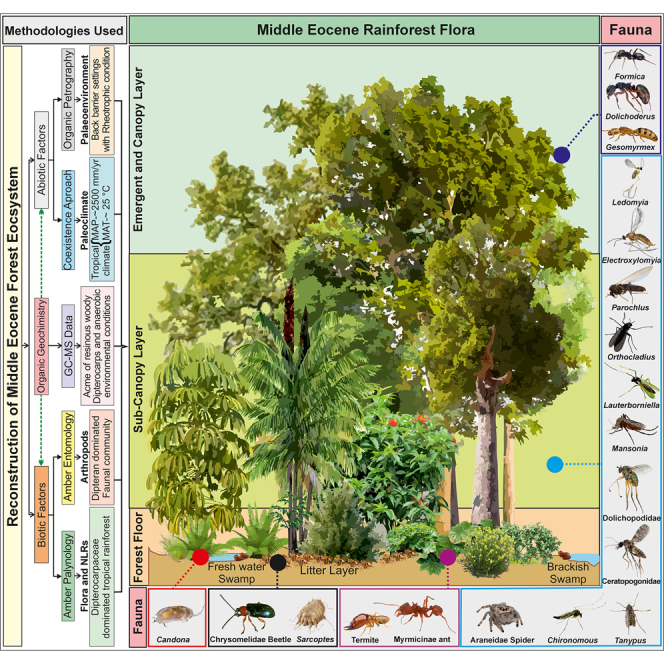

## Introduction

Tropical rainforests exemplify Earth’s most intricate and biologically diverse terrestrial ecosystems, hosting a disproportionate share of global biodiversity despite covering just ∼7% of the planet’s land surface.^1^ These ecosystems largely found in the equatorial Americas, Africa, and Indo-Pacific support nearly 50% of all known species.^2^ Their exceptionally high primary productivity coupled with intense interspecies interaction, establishes them as the “cradle” and “museum” of biodiversity.^3^^,^^4^ The exceptional diversity and ecological interactions have long fascinated evolutionary biologists, including Darwin, Bates, and Wallace, who regarded these ecosystems as ancient evolutionary crucibles, and are central to theories explaining the origins and persistence of biodiversity.

One such framework, the ESAT (energy-stability-area-time) theory, attributes high tropical diversity to elevated solar energy input, ecological stability over geological timescales, expansive historical distribution, and evolutionary time.^5^^,^^6^ Warmer, equable temperatures result in more species generations each year, with concomitantly higher rates of genetic variation and opportunities for both adaptive and phyletic evolution.^5^ The ecological stability of broad-leaved tropical forests particularly in terms of their taxonomic composition over geological timescales has likely played a key role in reducing extinction rates. These ecosystems have generally remained buffered from severe seasonality, glaciation, extreme droughts, tectonic upheaval, and other large-scale geophysical stresses.^7^^,^^8^ However, direct evidence of biodiversity in early tropical forests remains limited, particularly from low-latitude regions. The “area” variable within the ESAT model may seems counterintuitive, given the present-day restricted distribution of tropical forests. However, it is inextricably linked to the other variables, including *Time*. Angiosperm-dominated tropical rainforests commonly referred to as “megathermal” forests,^9^ appear to have existed since at the least early Paleocene (∼65 Ma),^10^^,^^11^^,^^12^ based on fossil records from the Western Hemisphere and the Maastrichtian-early Paleocene (late Cretaceous-early Paleocene) of the Southern Hemisphere.^8^^,^^13^^,^^14^

The Paleotropics, comprising about 50% of the world’s tropical rainforests coverage, host several biodiversity hotspots across Africa and Asia.^9^^,^^15^ India offers a compelling case study due to its dynamic geological history and rich fossil record.^16^ Currently, approximately 33% of all angiosperm species and 28% of all plant species in India are endemic.^17^ Interestingly, during the late Maastrichtian, India supported a highly endemic flora, with around 40% of its fossil floral assemblage being regionally unique.^8^ These assemblages are strikingly modern in composition and more taxonomically diverse than contemporaneous floras from the Northern and Southern Hemispheres.^8^^,^^18^ Such evidence positions India as a promising region for understanding the deep-time evolution of tropical rainforest biodiversity.^8^^,^^19^^,^^20^^,^^21^^,^^22^^,^^23^^,^^24^

The existing tropical rainforests of India are largely limited to the Western Ghats, northeastern states, and the Andaman and Nicobar Islands.^25^ However, from the Late Cretaceous (center of the Indian Plate was positioned at ∼20°S) to the Eocene (∼5°N), the Indian Plate migrated across the equatorial humid belt. This latitudinal shift facilitated the expansion of tropical ecosystems, which became increasingly widespread and covered large parts of western, southern, eastern, and central India.^8^^,^^21^^,^^26^^,^^27^^,^^28^ A low global temperature gradient during the early Paleogene enabled persistent warm and humid tropical conditions across much of the Indian Plate.^29^ As global temperatures rose and the Indian Plate continued its northward trajectory during the Eocene, these conditions intensified, leading to the development of luxuriant, multi-tiered rainforests and extensive peat swamps.^21^^,^^26^^,^^29^ Over time, the accumulated peat in these swamps lithified into lignite and coal deposits, particularly in western and northeastern India. Palynological studies conducted in these sedimentary deposits have enabled high-resolution biostratigraphy; phylogenetic analysis; paleobotanical, climatic, and environmental reconstructions.^7^^,^^22^^,^^30^^,^^31^^,^^32^^,^^33^ Uniquely, among all the other sedimentary deposits worldwide, western Indian lignite mines contain extraordinarily rich deposits of fossiliferous amber. These ambers serve as an exceptional archive of fossils with microscopic morphological details of biological inclusions, enabling unparalleled comparisons with myriad extant taxa.

The Umarsar Lignite Mine (ULM) in western India is a particularly important locality, as the stratigraphy of this mine is well established and preserves diverse fossilized biota in copious amber deposits. Thus, the ULM is an ideal site for understanding biotic communities, climate dynamics, and the ecological context of tropical forests during the Eocene epoch—a period characterized by global climatic instability and significant evolutionary turnover. This study focuses on the palynological and arthropod assemblages from ULM to reconstruct the biotic composition of the local middle Eocene ecosystem. The nearest living relative (NLR) method is employed to infer ecological affinities among fossil taxa; and the coexistence approach (CA), combined with organic geochemistry and petrographic data, is used to constrain key abiotic variables, such as paleoclimate and depositional environment. Our findings provide critical insights into how ancient tropical systems responded to past climate change and inform projections for the future of modern rainforests.

### Geological and stratigraphical background

**Figure 1:**
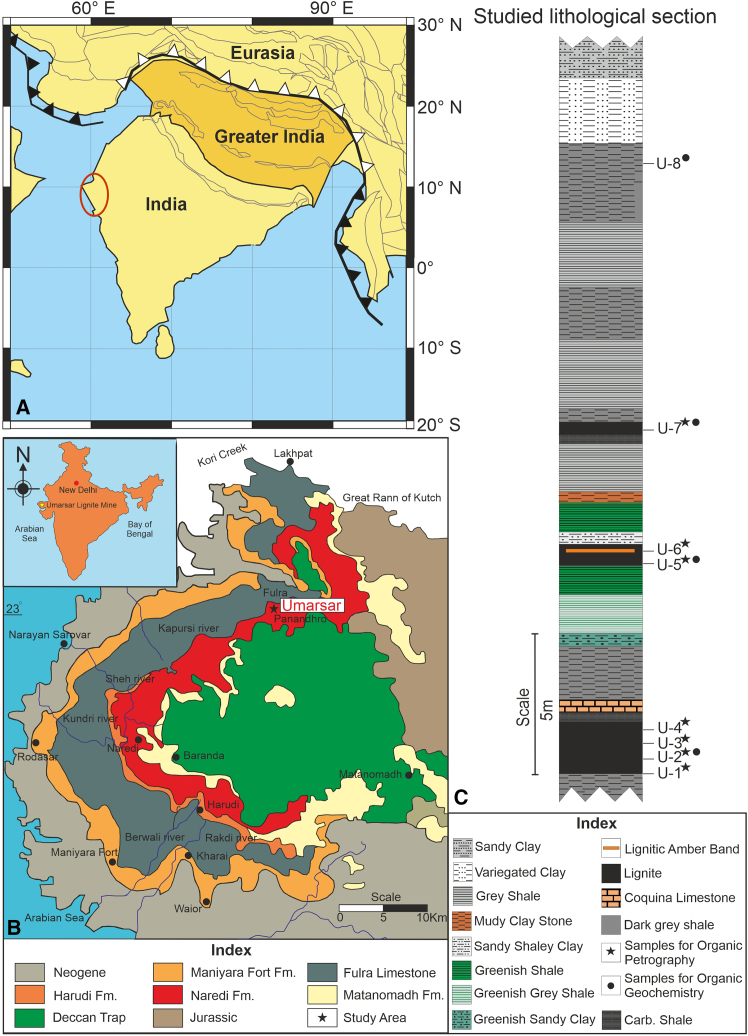
The paleogeographic and detailed geological map of the studied area (A) Paleogeographic map of the Indian Plate during the middle Eocene Plate reconstructed using GPlate and referenced from several van Hinsbergen et al.^42^; Müller et al.^35^; Westerweel et al.^43^ The red circle indicates the palaeogeographic position of western lignite mines, including Umarsar Lignite Mine. (B) Geographical map of the Umarsar Lignite Mine (ULM), Kutch. (C) Lithological characterization of the studied section from the Umarsar Lignite Mine, Kutch.

**Figure 2:**
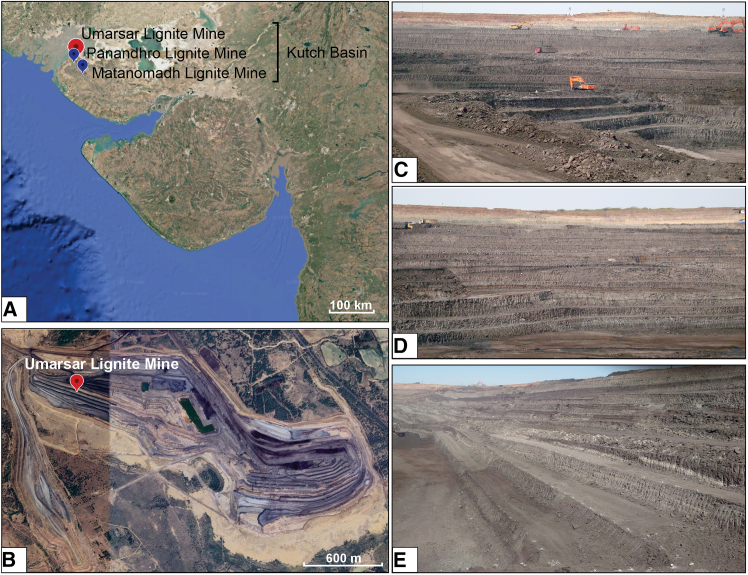
Location map of the Kutch Basin and Umarsar Lignite Mine, India (A) Distribution of lignite mines in the Kutch basin, Gujarat. (B) Location of the Umarsar Lignite Mine. (C–E) Outcrop section of the studied section in the Umarsar Lignite Mine.

The Kutch basin, located in western India, is a pericratonic rift basin formed during Gondwanan breakup that features a significant accumulation of Paleocene-Eocene strata, lignite, and fossiliferous amber deposits over the peneplained Deccan Traps (Figures 1 and 2,^34^). The studied sedimentary sequence occurs in the ULM, situated on the northwestern edge of the Kutch basin in Gujarat (latitude 23° 43′ 22.97″ N; longitude 68° 50′ 23.82″ E; Figures 1A and 2A). The sequence corresponds to the Harudi Formation, which is positioned at a paleolatitude of ∼10.5° N (paleolatitudes reconstructed using GPlates^35^; Figures 1B and 2B^34^^,^^36^). The lignite deposits of the Harudi Formation in the ULM are stratigraphically coeval with the adjacent Panandhro Mine, exhibiting no unconformity.^34^^,^^36^ The base of the Harudi Formation dates to the Lutetian-Bartonian boundary (∼41.6 ± 0.5 Ma), whereas its top has been confirmed as Bartonian (∼40.8 ± 0.5 Ma). The ages were inferred using strontium isotopes from the Coquina Bed, foraminiferal biomarkers, such as *Nummulites*, along with dinocysts.^34^^,^^37^^,^^38^^,^^39^ The deposition of the Harudi Formation has been correlated with the Kirthar transgression event, which occurred along the western margin of the Indian subcontinent.^40^ During this interval, marine transgression in the ULM created marginal to shallow marine environments.^41^ The studied sedimentary succession consists primarily of shale and sandy clay, interspersed with layers of loose amber beds containing medium-sized nodules (Figures 1C and 2C–2E).

## Results

### Paleoclimate estimates for the middle eocene in western India

**Figure 3:**
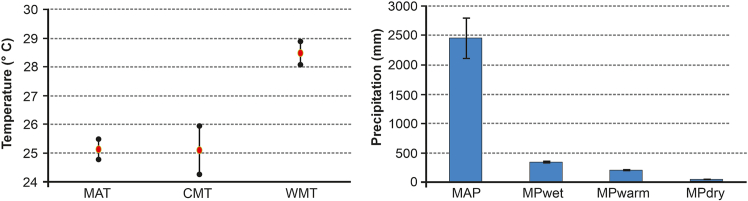
Paleoclimatic reconstruction for the middle Eocene based on the coexistence analysis of the studied Umarsar Lignite Mine floral assemblage Figure relating to Data S1.

The CA is a widely adopted methodology in paleoecology for reconstructing paleoclimate using climate data from NLRs.^44^^,^^45^^,^^46^^,^^47^ The paleoclimate estimate indicates a megathermal climate during the middle Eocene (Figure 3 and Data S1).

### Temperature

**Figure 4:**
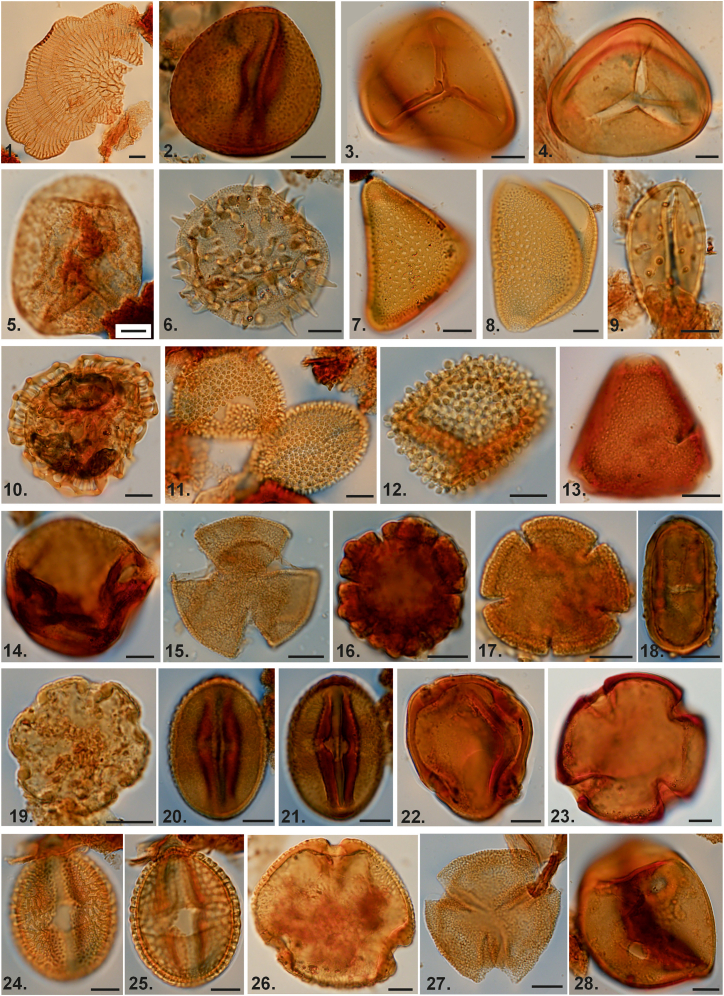
Photomicrographs of characteristic palynomorphs from the Umarsar Lignite Mine, Harudi Formation, Gujrat 1. Fungal thalli, BSIP Museum Slide no. 17474, L41/2; 2. *Acrostichum meghalayaensis* Kar, 1991, BSIP Museum Slide no. 17479, N16/3; 3. *Cyathidites congoensis* Sah, 1967, BSIP Museum Slide no. 17474, S28/1; 4. *Dandotiaspora dilata* Sah et al., 1971, BSIP Museum Slide no. 17474, E27/3; 5. *Araucariacites australis* Cookson, 1947, BSIP Museum Slide no. 17480, W32/2; 6. *Spinizonocolpites bulbospinosus* Singh, 1990, BSIP Museum Slide no. 17481, M34/4; 7. *Longapertites triangulatus* Samant and Phadtare, 1997, BSIP Museum Slide no. 17475, M30/3; 8. *Longapertites hammenii* (Rao and Ramanujam) Rao, 1990, BSIP Museum Slide no. 17475, H25/2; 9. *Arengapollenites ovatus* Kar and Bhattacharya, 1992, BSIP Museum Slide no. 17464, E45/2; 10. *Retipollenites confuses* Gonzalez Guzman, 1967, BSIP Museum Slide no. 17475, H25/1; 11. *Incrotonipollis vastanensis* Bansal et al., 2022, BSIP Museum Slide no. 17474, S8/4; 12. *Incrotonipollis indicus* Bansal et al., 2022, BSIP Museum Slide no. 17463, X24/4; 13. *Proteacidites* sp., BSIP Museum Slide no. 17475, K31/4; 14. *Corsinipollenites jussiaeensis* Jan du Chene et al., 1978, BSIP Museum Slide no. 17473, G22/3; 15. *Dipterocarpuspollenites retipilatus* Kar, 1992, BSIP Museum Slide no. 17480, V26/4; 16. *Ctenolophonodites costatus* (von Hoeken-Klinkenberg) von Hoeken-Klinkenberg, 1966, BSIP Museum Slide no. 17482, M35/1; 17. *Polybrevicolporites cephalus* Venkatchala and Kar, 1969, BSIP Museum Slide no. 17473, O20/2; 18. *Umbelliferoipollenites ovatus* Venkatchala and Kar, 1969, BSIP Museum Slide no. 17462, M31; 19. *Pseudonothofagidites kutchensis* Venkatachala and Kar 1969, BSIP Museum Slide no. 17462, N21/4; 20. and 21. *Lagerstroemia cathayensis* Lieu et al., 2008, BSIP Museum Slide no. 17468, F16/2; 22. *Meliapollis minutus* (Singh) Singh, 1990, BSIP Museum Slide no. 17466, U16/3; 23. *Meliapollis ramanujamii* (Sah and Kar) Rao, 1990, BSIP Museum Slide no. 17481, K11/4; 24. and 25. *Horniella* sp., BSIP Museum Slide no. 17483, O18/1; 26. *Pellicieroipollis langenheimii* Sah and Kar, 1970, BSIP Museum Slide no. 17481, L31; 27. *Sastripollenites trilobatus* Kar, 1978, BSIP Museum Slide no. 17470, K40/2; 28. *Lakiapollis ovatus* Venkatchala and Kar, 1969, BSIP Museum Slide no. 17463, R25. Scale =10 μm.

The Eocene was the warmest interval of the Cenozoic era, with significantly elevated mean annual, cold month and warm month temperatures compared to current values.^29^^,^^48^ This period was defined by reduced temperature gradients,^29^^,^^49^ which fostered an exceptional proliferation of tropical flora and fauna into extratropical regions, referred to as the “Eocene Paradise”.^50^ The findings of the CA reveal that the middle Eocene experienced frost-free, warmer climates with minimal seasonal temperature variability. The reconstructed paleoclimate illustrates a mean annual temperature (MAT) of 25.15° ± 0.35°C, a cold month mean temperature (CMT) of 25.1° ± 0.8°C, and a mean warm month temperature (WMT) of 28.5° ± 0.4°C (Figure 3). These climatic estimates align with the paleolatitudinal position of the Indian Plate (∼10.5 °N; Figure 1A). The palynoassemblage is dominated by a diverse range of megathermal angiosperm families (see in the following text), indicating a consistently warm and moist tropical climate (Figures 4 and S1; Table S1 for complete list).

### Precipitation

In general, temperature and precipitation are positively correlated, with an increase in temperature corresponding to a proportional rise in precipitation.^51^ During the middle Eocene, the climate around the Umarsar Lignite Mine was predominantly humid, characterized by a high mean annual rainfall (MAP) of 2455.5 mm ± 244.5 mm, which was seasonal (Figure 3), with a significant difference between the mean rainfall during the wettest month (MPwet: 348 mm ± 2 mm) and the rainfall during the driest month (MPdry: 39.5 mm ± 3.5 mm). Additionally, the warmest month (MPwarm) saw substantial precipitation with 213.5 mm ± 7.5 mm of rainfall (Figure 3).

**Figure 5:**
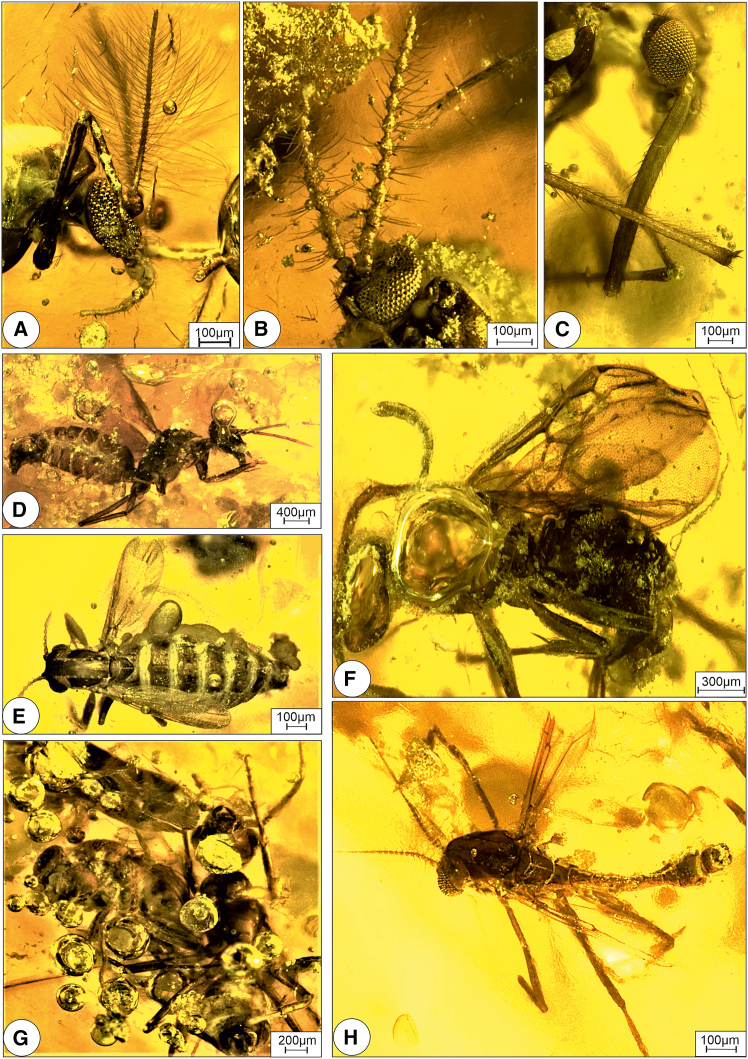
Arthropod assemblage embedded in Kutch Amber from the Umarsar lignites Light microscopy images of (A) Orthocladinae indet, BSIP museum no. 42213; (B) *Ledomyia* sp., BSIP museum no. 42215; (C) *Mansonia* sp., BSIP museum no. 42218; (D) *Formica* sp., BSIP museum no. 42219; (E) Ceratopogonid midge, BSIP museum no 42216; (F) *Dolichoderus* sp., BSIP museum no. 42223; (G) *Gesomyrmex* sp., BSIP museum no. 42220 and (H) Podominae indet, BSIP Museum no. 42212. Figure relating to Table S3.

Considering the paleolatitude of the studied section at ∼10.5° N (Figure 1A), it is likely that Indonesian-Australian-type monsoon-like conditions were prevailing during the middle Eocene.^52^^,^^53^ The high levels of monsoonal rainfall were likely influenced by the proximity of the studied section to the adjacent ocean (Figures 1A and 2A). During the Eocene, stable warm conditions in the tropics and a global sea surface temperature of about 32.5 ± °C generated a significant thermal gradient between land and ocean, leading to abundant seasonal rainfall.^29^^,^^54^ The prevalence of certain plant and arthropod families reflect the existence of wet and seasonal conditions (Figures 4 and 5 and Tables S1–S3).

### Paleobiota and paleoenvironment

**Figure 6:**
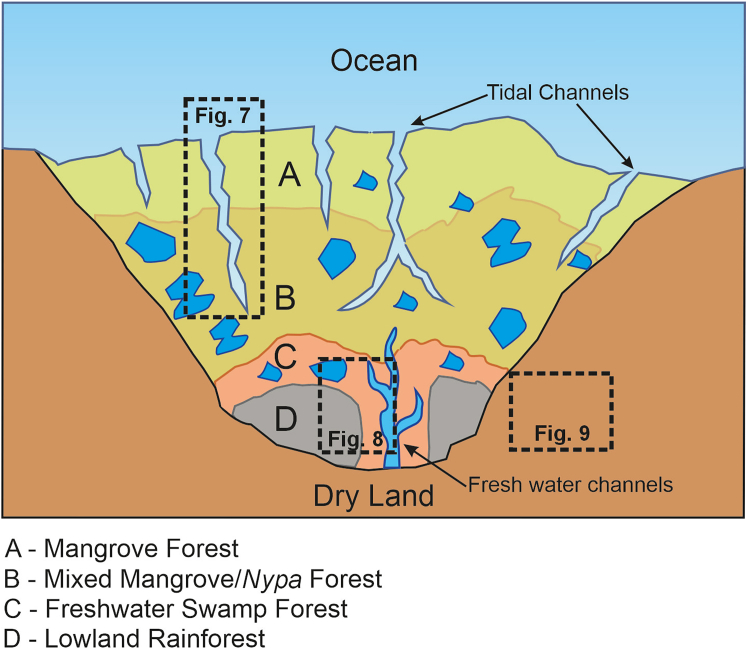
Schematic diagram showing distribution of different floral communities across the landscape Figure relating to Table S1.

Details of the arthropod community (Figures 5 and S2; Tables S2 and S3), paleofloristic composition and paleoenvironmental conditions (Figure 3; Table S1), along with petrographical (Table S4) and organic geochemical data (Tables S5–S7) from amber inclusions and sediments, collectively indicate the presence of tropical rainforest ecosystem during the middle Eocene, characterized by a heterogeneous mosaic of habitats (Figure 6). Approximately 85% of the identified flora is composed of tropical wet evergreen forest taxa, followed by subtropical (8%), temperate (6%), and tropical moist deciduous taxa (1%; Figure S1). The floristic assemblage is dominated by woody angiosperms, which account for 68% of the total plant diversity (75 taxa), whereas herbaceous angiosperms represent approximately 11% (12 taxa; Figure S1). Pteridophytes (ferns) and gymnosperms are represented by 10 and one taxon, respectively, while the remaining fraction comprises unknown types (Table S1).

The dominance of woody vegetation is also highlighted by moderate concentrations of ulminite (huminite) maceral (Figure S3; Table S4) with P_wax_ ratios ranging from 0.57 to 0.75 (Table S5), indicative of significant higher-plant input. The low inertinite content, combined with limited representation of semifusinite macerals, suggest deposition under predominantly anaerobic conditions.^55^^,^^56^ Episodes of quick and brief subsidence in water levels may have facilitated effective preservation of organic matter. Consequently, a well-preserved and taxonomically diverse flora has been recovered, with most of the NLRs belonging to lowland paleotropical angiosperm families, such as Arecaceae^33^^,^^57^ Dipterocarpaceae,^22^ Fabaceae,^58^ Euphorbiaceae,^59^ Ctenolophonaceae,^60^ Proteaceae,^61^ and Sapotaceae.^62^ These floral associations reflect strong ecological and compositional parallels with extant paleotropical rainforests.^9^^,^^63^ Sediment analysis further reveals a bimodal distribution of *n*-alkane series (*n*-C14–*n-*C33), indicative of mixed inputs from terrestrial and aquatic organic matter (Figure S4).

**Figure 7:**
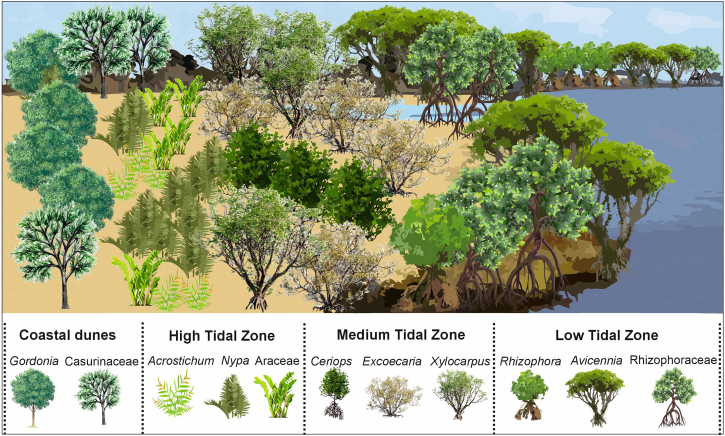
Simplified landscape model of coastal mangrove biotic community Figure not to scale and relating to Table S1.

The preserved biota originates from vegetation thriving in a complex heterogeneous landscape, comprising muddy coastal intertidal swamps with episodic brackish water influxes, freshwater back-swamps, stratified lowland tropical rainforests, montane and riparian forests, and open-canopied ecotonal regions (Figure 6). The coastal swamp community includes thirteen taxa associated with halophytic mangrove and coastal environments (Figure 7). The presence of framboidal pyrite in the stratigraphic section indicates marine transgression in the mire system (Figure S3).^64^^,^^65^^,^^66^ Additionally, coalification indices, such as the gelification index (GI) and tissue preservation index (TPI) further signify deposition of limno-telmatic vegetation in a back-barrier swamp environment (Figure S5). The detection of dinoflagellate cysts at multiple depth intervals within the sedimentary sequence of the Umarsar Lignite Mine^67^ also advocates episodic marine transgression into the mire system.

**Figure 8:**
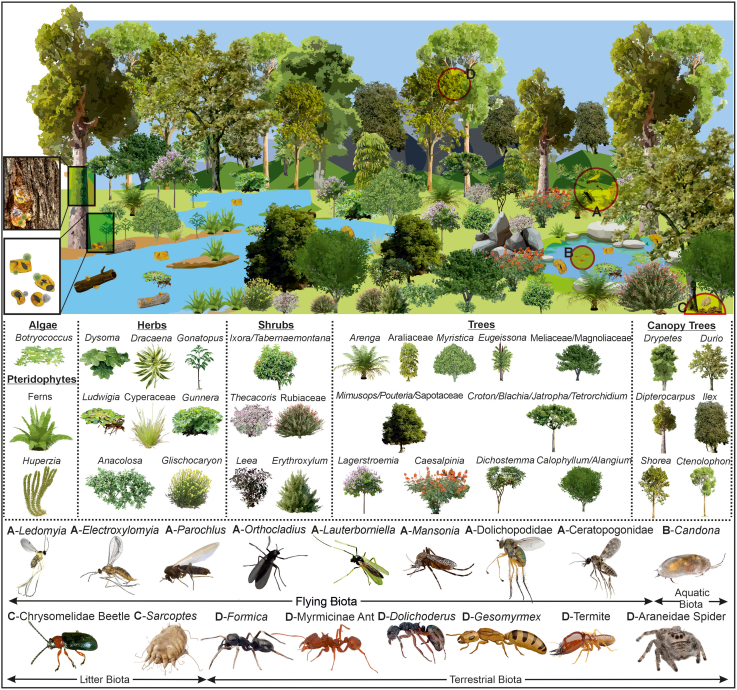
Simplified landscape model of lowland rainforest and fresh water swamp biotic community Figure not to scale and relating to Table S1.

Further inland, a transition from coastal swamps to freshwater-dominated environment is inferred encompassing lake margins, large river basins, deltas, floodplains, and low-elevation hill slopes and valleys (Figure 8). The groundwater index (GWI) and vegetation index (VI) indicate rheotrophic conditions across marginal-aquatic to swamp settings, inhabited by a mixture of arborescent and herbaceous vegetation (Figure S6). During the middle Eocene, these habitats hosted dense multistoried tropical forest vegetation (Figure 8) supporting high biodiversity by offering a wide range of ecological niches and microhabitats. The diverse vegetation structure sustained a broad spectrum of arthropod communities, including Formicidae (ants), Coleoptera (beetles), Araneae (spiders), Isoptera (termites), and various Dipteran groups, such as bitting, non-biting, and gall midges (Figure 8; Table S2).

**Figure 9:**
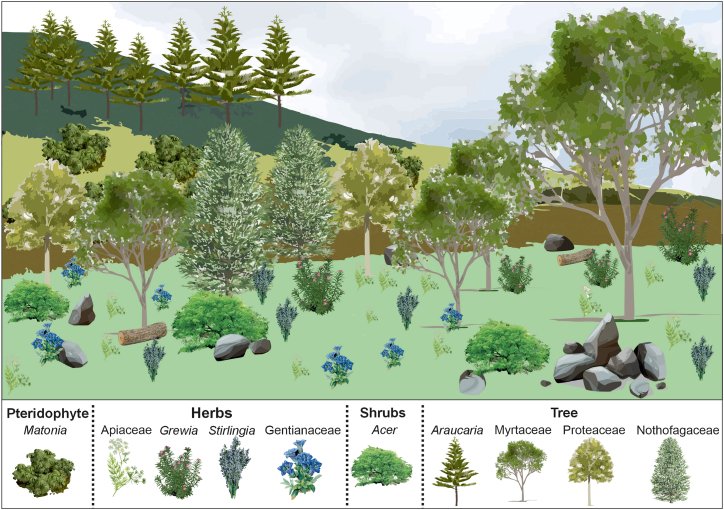
Simplified landscape model of forest outlier and upland biotic community Figure not to scale and relating to Table S1.

In ecotonal zones, lowland tropical rainforest vegetation has fragmented into open-canopied, sparsely vegetated patches (Figure 9). These transitional habitats were dominantly occupied by small trees, shrubs, and herbs, with nearby hinterlands hosting temperate pteridophytes and conifers adapted to higher terrain and microclimates.

## Discussion

### Paleoecological reconstruction

Paleoecological reconstruction fundamentally relies on uniformitarianism, assuming that the ecological roles and behavioral attributes of extinct organisms were analogous to those of their NLRs.^46^ Our investigation of the Umarsar Lignite Mine revealed an exceptionally diverse fossil assemblage, including over 800 arthropod specimens spanning multiple taxonomic ranks (Table S1), along with 78 genera and 118 species of palynomorphs (>100; Data S1). Although our paleobiotic reconstruction is informed primarily by the flora, selected arthropod taxa analyzed in detail herein have also been integrated into our reconstruction (Table S3). The recovered biota supports a megathermal monsoonal paleoclimate with a heterogeneous landscape comprising three distinct floristic communities (Figure 6), discussed in the following text.

#### Coastal mangrove forests

Mangrove forests typically dominate low-wave energy coastlines and intertidal habitats in tropical and subtropical regions (Figure 7). Paq (proxy aqueous) values in our samples (0.54–0.57; Table S5) confirm the contribution of organic matter from emergent aquatic vegetation, particularly mangroves. Mangrove taxa, such as *Rhizophora* sp. (Rhizophoraceae) and *Avicennia* (Acanthaceae) and other members of the Rhizophoraceae were dominant in low tidal zones, forming dense fringing mangrove forests. In tidally influenced, landward zones, *Ceriops* (Rhizophoraceae), *Excoecaria* (Euphorbiaceae), and *Xylocarpus* sp. (Meliaceae) likely formed mixed mangrove communities.

Further inland, brackish swamps were colonized by plants, such as *Acrostichum*, *Nypa*, and members of Araceae, taxa known for their ecological plasticity across salinity gradients (Figure 7). *Nypa fructicans*, in particular, is highly salt-tolerant and dominates tidal swamp ecosystems in modern New Guinea’s mangrove forests.^68^ Beyond the influence of tidal inundation, sandy coastal habitats supported taxa, such as *Gordonia* (Thecaceae) and Casuarinaceae trees. The Umarsar amber forest shares strong compositional similarity with extant mangrove communities, reflecting the prevalence of Rhizophoraceae, Acanthaceae, Meliaceae, Euphorbiaceae, and Arecaceae (*Nypa*).^69^

#### Lowland rainforest and freshwater swamp vegetation

Forest structure is governed by four key ecological parameters: climate, floristic composition, vegetation stratification, and plant physiognomy. Lowland tropical rainforests, typically grow under high-mean annual precipitation (>1800 mm yr-1), elevated mean annual temperatures (18–28°C), and minimal temperature seasonality (<7°C range), supporting taxonomically diverse families and a dominance of angiosperms. Similar fossil evidence from the Umarsar Lignite Mine points to a dense-canopied, hyper-diverse lowland tropical rainforest with distinct vertical stratification. These rainforests likely extended across vast swampy lowlands and foothill zones (Figures 6 and 8). The flora is dominated by megathermal families, such as Dipterocarpaceae, Fabaceae, Euphorbiaceae, and Arecaceae, exhibiting compositional similarity with extant southeast Asian forest.^63^ Tropical rainforest stratification typically comprises four layers: the emergent (>40 m), high upper canopy (30–40 m), lower sub-canopy; shrub understory, and ground layer. Palynological data reveal the presence of broad-leaved hardwood and evergreen taxa, such as *Dipterocarpus*, *Shorea*, and *Durio* in the emergent layer, with *Dipterocarpus* being the most abundant (Figure 8). These resin-producing trees thrive in lowland areas near riparian fringes and coastal hills, requiring prolonged wet periods (≥8 months). Their prevalence is also corroborated by terpenoid biomarkers—germacrene-D derivatives, such as muurolene, 5, 6, 7, 8-tetrahydrocadalene, calamenene, dihydro-ar-curcumene, and cadalene (Figure S7 and Table S6).^70^ The abundance of resinite macerals in the sediment samples also signifies the dominance of dipterocarps in the arboreal forest vegetation (Table S4). Today, dipterocarps comprise up to 80% of the canopy-emergent trees in Southeast Asia,^71^ with Borneo representing the center of extant diversity (∼287 species in 9 genera), in contrast to only 34 species in Africa and Madagascar. Despite this disparity, the paleobotanical evidence suggests that the family originated in Africa and diversified in India, before dispersing to Southeast Asia, where it established the lowland dipterocarp forest during late Eocene.^22^ In the absence of wood-boring arthropods commonly associated with resin induction in modern trees (e.g., bark beetles: Scolytinae and Platypodinae), the formation of ULM amber may instead reflect extensive mechanical damage caused by intense monsoonal events.

Below the emergent layer, the upper canopy comprised trees, such as *Ctenolophon*, *Drypetes*, and young trees of emergent species. Tall palms (Arecaceae) also occupied this stratum. A similar association of *Ctenolophon* trees with dipterocarps as canopy trees is observed in the undisturbed lowland mixed dipterocarp and peat swamp forests of the southeast Asia (up to 900 m altitude).^9^ These arboreal canopies provided niches for the terrestrial arthropod fauna, particularly ants and termites (Figure 8). Ants attributed to Myrmicinae, Dolichoderinae, and Formicidae subfamilies, including *Dolichoderus*, *Formica*, and *Gesomyrmex*, formed social colonies in arboreal canopies or in other terrestrial environments (Tables S2 and S3). Diverse ant community belonging to Formicidae family and Myrmicinae subfamily, have been recovered in groups from the amber inclusions, suggesting that the castes of ants, including the queen, workers, and males, functioned as cohesive groups during the middle Eocene (Table S2).

The dense canopy of tropical rainforests significantly restricts light availability to the lower canopy and understory, creating a stratified light environment that shapes the composition and distribution of plant taxa. Consequently, these strata host a diverse assemblage of both shade-tolerant and shade-intolerant species. The shade-tolerant flora of Umarsar Lignite Mine includes shrubs, such as *Arenga*, *Eugeissona*, *Myristica*, *Dichostemma*, *Mimusops*, *Pouteria*, *Alangium*, alongside herbaceous taxa, such as *Erythroxylum*, *Anacolosa*, and *Gonatopus*, which thrive under low light conditions. Conversely, shade-intolerant species in the Umarsar Lignite Mine rainforest include trees and shrubs of *Lagerstroemia*, *Caesalpinia*, *Thecacoris*, *Croton*, *Blachia*, *Jatropha*, *Tetrorchidium*, and *Calophyllum*, along with understory herbs like *Ixora*, *Tabernaemontana*, *Leea*, *Glischocaryon*, and *Gunnera*. These species are typically fast-growing opportunists that rapidly colonize canopy gaps created by tree mortality, windthrow, or forest fires.^72^ Shade-tolerant seedlings and saplings, while slower-growing under closed canopies, experience rapid vertical growth when exposed to increased light availability in tree-fall gaps.

These floristic dynamics, coupled with vertical stratification, also create a mosaic of microhabitats in the sub-canopy and understory layers that support a diverse arthropod community. In this study, various taxa of beetles, flies, mosquitoes, and seven species of flying midges were identified as likely inhabitants of these layers (Figure 8). Notably, wasps, and butterflies were also present but are under-represented due to their avoidance of resin, limiting their potential for preservation in amber. Identified arthropod taxa include gall midges (*Ledomyia* and *Electroxylomyia*), non-biting midges, such as *Orthocladius*, *Parochlus*, *Lauterborniella*, *Chironomous*, and *Tanypus*, bitting midges (Ceratopogonidae), long-legged flies (Dolichopodidae), and a single mosquito taxon, i.e., *Mansonia* (Table S3). Gall midges induce gall formation in host plants for oviposition, with both larvae and adults feeding on fungal and plant tissue. The proliferation of fungi and plants in the tropical biome provided abundant food resources for these phytophagous arthropods.

Geochemical indicators, such as CPI, Paq and Pwax, and β-amyrin derivatives (oleanene compounds) suggest the presence of lacustrine and perennially moist habitats within the Umarsar Lignite Mine rainforest (Tables S5 and S7). These swampy environments supported a diverse pteridophyte community incorporating Schizaeaceae, Dipteridaceae, Filicales, Dennstaedtiaceae, and Osmundaceae (Table S1). Some ferns of the families Osmundaceae, Dipteridaceae, and Schizaeaceae may have been epiphytic, paralleling their ecological roles in modern tropical forests.^73^^,^^74^^,^^75^ The abundance of detrohuminite in the telohuminite group of macerals further supports the input of organic matter from non-arboreal vegetation, including herbaceous plants, shrubs, and ferns (Table S4). Plants of various other tropical families, such as Arecaceae, Araceae, Fabaceae, Meliaceae, Magnoliaceae, Araliaceae, Sapotaceae, and Rubiaceae must have occupied variety of habitats within the tropical rainforest. Lowland swamp vegetation in the ULM included small trees of *Myristica*, *Gonatopus*, *Calophyllum*, *Dichostemma*, *Poteria*, *Tetrorchidium*, *Lagerstroemia*, *Croton*, and *Alangium* (Figure 8). Wetland-adapted taxa, such as *Myristica* and members of Cyperaceae (sedges) were particularly prominent. Additional evidence for the presence of freshwater bodies, such as ponds or oxbow lakes, comes from the occurrence of *Botryococcus* (a green alga), pollen of the aquatic angiosperm *Ludwigia*, and the freshwater ostracod *Candona.* These swampy habitats not only supported a rich vegetal community but also provided breeding grounds and refuge for the larvae of *Mansonia* mosquitoes and various biting and non-biting midges. Within these aquatic ecosystems, *Candona*, thrived on freshwater detritus and diatoms, highlighting the ecological complexity and biodiversity of the ULM swamp rainforest ecosystems.

Just as tropical rainforest plants compete intensely for light above ground, below ground they vie for mineral nutrients, as tropical rainforest soils are typically nutrient poor.^76^ In the ULM rainforest, evidence of rapid nutrient recycling is reflected by low coal content indices of CPI (0.8–1.26), TPI (<1), and Paq (0.32–0.56), along with a relative dominance of the densinite maceral (Table S2).^77^ These indicators collectively point to accelerated organic matter decomposition and nutrient turnover. This rapid nutrient cycling is primarily mediated by microbial communities, particularly bacteria and fungi, which are essential for maintaining forest health. The abundance of funginite (9.1%) within the inertinite maceral group, alongside well-preserved fungal structures such as Microthyriaceae thalli in amber and sediment samples, supports the existence of a rich fungal assemblage in the ULM forest (Table S4).^77^^,^^78^ In addition to microbial and fungal decomposers, arthropods particularly termites play a pivotal role in nutrient cycling. Termites can decompose over half of the deadwood biomass in modern tropical rainforest.^79^ Although termite representation in the ULM assemblage is limited to a single taxon within the subfamily Termitidae, their ecological role in wood decomposition was likely significant. The enormous litter production in tropical rainforests also provides critical microhabitats for various litter-dwelling arthropods, including mites and beetles. Representative taxa, such as the *Sarcoptes* mite and members of the Chrysomelidae beetle family were recovered from ULM deposits (Figure 8). *Sarcoptes kutchensis* appears to have been highly specialized, feeding on melanized ectomycorrhizal fungi (Dematiaceae), indicative of a trophic link with specific soil fungal communities.^80^^,^^81^ In contrast, Chrysomelid beetles encompass a broader ecological range, engaging in folivory, nectivory, and detritivory.^82^

Vegetation in slightly elevated or hilly areas of the ULM rainforest included lycophyte, such as *Huperzia*, herbaceous taxa, such as *Dracaena*, *Dysoma*, and *Erythroxylum*, and tree species including *Eugeissona* and *Ilex* (Figure 9). These taxa represent a distinct assemblage, potentially associated with well-drained, upland microhabitats.^83^

#### Ecotonal outlier flora

In addition to the tropical rainforest flora, the ULM also featured open canopy ecotonal regions, where subtropical, temperate, and deciduous elements intermixed with tropical flora. These transitional zones likely formed a mosaic of habitats ranging from sparsely forested to semi-open landscapes, reflecting environmental gradients and climatic variability (Figure 9). The ecotonal vegetation was dominated by herbaceous taxa, such as *Stirlingia*, *Grewia*, and members of Apiaceae and Gentianaceae, together with *Acer* shrubs. Tree taxa from Myrtaceae, Proteaceae, and Nothofagaceae suggest the presence of slightly drier forest margins or seasonal habitats adjacent to the tropical rainforest core (Table S1). Open-adapted ferns such as *Matonia*, typically found in mossy mountain summits today, likely colonized higher altitudinal zones within the hinterland. The recovery of buoyant pollen grains of *Araucaria* implies that some of the organic material was transported from distant montane regions, contributing to the palynological and maceral assemblages of the ULM lowland system (Figure 9).^84^

### Arthropods of the ULM

An arthropod fossil assemblage so far recovered from the amber inclusions of ULM comprises over 830 specimens of terrestrial arthropods belonging to at least 45 families in 25 orders (Table S2). The assemblage is dominated by surface-dwelling taxa, including both wingless forms (arachnids, apterygote hexapods, worker ants), and weak fliers that exist in microhabitats, such as leaf litter, bark crevices, and plant surfaces (e.g., Blattodea [including termites], Psocodea, Heteroptera, and Coleoptera)^85^^,^^86^ A broad spectrum of arthropod ecological guilds represented in the ULM amber, underscores the high biodiversity and trophic complexity of the Middle Eocene rainforest ecosystem (Tables S1 and S2). Predators include spiders (Araneae), predatory millipedes, staphylinid beetles (Staphylinidae), dolichopodid flies (Dolichopodidae), and various ant species (Formicidae). Scavengers are represented by dermestid beetles (Dermestidae) and phorid flies (Phoridae), whereas parasitoids are particularly diverse, including members of at least eight families of non-formicid apocritan wasps and possibly some phorid flies, all indicative of a well-structured trophic web with a rich diversity of available hosts.

Among pollinators, the most significant were *Melikertes* bees, a now-extinct lineage of stingless bees (Meliponini) that persisted from the Eocene (Baltic region, and herein) through the Eocene-Oligocene extinction event.^87^ Meliponines are disproportionately preserved in Cenozoic ambers due to their active collection of resin for nest construction. *Melikertes* bees serve as direct evidence for pollinator activity, but it is highly likely that other arthropods pollinators especially Diptera were also present but are under-represented due to preservation biases. Lepidoptera are particularly rare in amber, likely due to their easily shed scales which facilitate escape from sticky substrates, such as resin and spider webs.

Relatively few phytophagous groups are preserved in the ULM amber (Table S2). These include members of Hemiptera and several beetle families belonging to Chrysomelidae and Curculionidae families and Alticinae subfamily. In contrast, saprophagous and microbivores groups are notably diverse and abundant. Saprophagous include taxa associated with decaying wood (e.g., Isoptera, Elateroidea, and Mycetophilidae), as well as microbial grazers that feed on algal mats, fungal hyphae, yeast, and bacterial films.^88^^,^^89^^,^^90^^,^^91^ These “microbivores” include Collembola, Archaeognatha, Blattodea, Psocodea, Ptiliidae, and the larvae of at least nine families of nematoceran Diptera. Adult Chironomidae (non-biting midges) are particularly abundant in ULM amber, more so than in contemporaneous Cambay Basin amber (Table S2). Chironomid larvae are aquatic and feed on diatoms and algae through grazing or filtering mechanisms. Their abundance indicates nearby low energy, eutrophic freshwater environments, such as ponds or slow-moving streams.^92^^,^^93^ Their abundance suggests proximity to such freshwater bodies and argues against a perennially inundated swamp forest, which typically supports lower ant diversity. The co-occurrence of terrestrial ants and aquatic midges, thus, reflects a heterogeneous paleoenvironment with a mosaic of well-drained forest floors and adjacent aquatic habitats (Figure 8).

The recovered assemblage exhibits an over-representation of certain lineages, such as non-biting midges and ants, and an under-representation of others, including termites and mites, with a near absence of groups such as butterflies and moths. This disparity is likely attributable to a combination of biological, morphological, and taphonomic biases inherent in the fossilization process.^94^ The site and context of resin production play a crucial role in insect entrapment. Insects living in close proximity to resin-producing trees are most susceptible to entrapment. While small, airborne insects from more distant areas may occasionally be transported by wind into resin, such occurrences are less likely in a densely vegetated, multistoried tropical rainforest, similar to the presently reconstructed the middle Eocene rainforest (Figure 8).^95^^,^^96^ Insects inhabiting arboreal canopies, wet soil, leaf litter, bark crevices, and foliage are well represented in the studied faunal community, whereas taxa associated with more distant coastal (Figure 7) or ecotonal environments (Figure 9) are notably absent. Moreover, a discernible bias toward small-bodied arthropods is also observed, likely reflecting their higher probability of becoming entrapped in resin. Most preserved specimens are in the range of 1–3 mm in length, consistent with size distributions reported in Miocene Mexican amber.^86^

### Plant–arthropod interactions

Insects and angiosperms engage in a diverse ecological interaction, ranging from mutualism to antagonism.^97^ These relationships between both groups likely ancestral and have played a crucial role in the evolutionary diversification.^98^ Given the incredible diversity of both groups, their interactions hold substantial ecological significance for the structure and functioning of terrestrial ecosystems.^99^^,^^100^ The simplest interaction between angiosperms and insects is antagonistic. Nearly 50% of insect species are known to consume plant tissues and their by-products. Similarly, the studied assemblage primarily consists of phytophagous insects, including ants, non-biting midges, gall midges, beetles, termites, and mites. These insects exhibit varied food preferences, with midges and mites primarily consuming decaying forest litter, while other taxa feed on living plant materials (e.g., ants, gall midges, beetles, and termites). They also differ in feeding specialization, ranging from monophagous (e.g., gall midges) to oligophagous (e.g., beetles) and polyphagous (e.g., ants and termites; Figure 8).^101^ Among them, *Ledomyia* and *Electroxylomyia*, two gall midges, from the Cecidomyiidae family, exhibit extreme antagonistic behavior by not only feeding on plant tissues but also causing plant tissue malformations known as galls. In contrast, non-biting midges, termites, and *Sarcoptes* mites form a commensal relationship with plants, consuming organic detritus, including decaying leaves and other plant matter. Interestingly, non-biting midges and *Sarcoptes* mites have evolved parasitic interaction with mammals over time.^80^ Moreover, the fossils of Dolichopodidae, Ceratopogonidae, and *Mansonia* mosquitoes from the middle Eocene suggest parasitic or commensal associations with vertebrate predators and potential pest-host interactions. The long-legged flies of Dolichopodidae family are predators that feed on other small arthropods like aphids, bark lice, etc.^102^^,^^103^ Meanwhile, biting midge and mosquitoes are hematophagous, likely feeding on the blood of various vertebrates present during the middle Eocene, including crocodiles,^104^ rodents,^105^ snakes,^106^ and mammals^107^^,^^108^ etc.

Eusocial insects, such as ants and termites, are also identified in studied amber inclusion. The ecological significance of ants is imperative and they serve as keystone taxa in numerous tropical forest habitats.^109^ In the present study, ants were observed to occupy different layers within the forest. For instance, farmer ants like *Dolichoderus*, *Formica*, and *Gesomyrmex* create leaf nests in the canopy and sub-canopy layer. While, the myrmicines are hyperdiverse clade which constitute diverse array of socially parasitic species.^110^ Among all the most notable the iconic mutualism between fungus-growing ants and their cultivated fungi.^111^ The underground chambers and galleries in the nests of Myrmicinae ants and termites maintain nutrient recycling and hydrology of tropical rainforests, establishing them as key ecosystem engineers.^112^ Termites, in particular, are responsible for decomposing over half of the deadwood in tropical rainforests, significantly causing wood mass loss.^79^ This process of wood loss creates canopy gaps, mediating another critical ecology of tropical rainforest ecosystems for maintaining biodiversity within these ecosystems. The recovered faunal assemblage, comprising various ants and a termite, reflects the trophic complexity and ecological interactions within the middle Eocene Forest ecosystem.

Among the various trophic interactions between plants and insects, the mutualistic relationship stands out as the most significant in contemporary ecosystems. This ancient interaction confers reciprocal benefits, with insects acquiring food, shelter, and protection, and plants relying on insects for seed dispersal and pollination. Such mutualistic relationships are essential for ecosystem health and are believed to have driven the co-diversification of both communities in the tropics.^113^ Evidence of these interactions becomes increasingly prominent from the Eocene onwards.^113^ Ants are key to seed dispersal, while various insect groups such as bees, flies, wasps, moths, butterflies, beetles, and thrips play a crucial role in pollination. Approximately 70% of angiosperms globally rely on insects for pollination, a figure that rises to 75% in tropical regions.^100^ Likewise, over 80% of taxa in the studied assemblage are pollinated by various faunas (Figure S10), with over 55% relying specifically on insects, 15.2% on different fauna (including insects) and 7.6% exclusively on vertebrates. Wind pollination is more prevalent in open habitats, resulting in only 2.2% of reported taxa being wind-pollinated in the studied assemblage (Figure S10). Bees, moths, and beetles are among the principal pollinators of the tropical rainforest. Notably, most of these groups are absent from the ULM amber, likely due to their behavioral aversion to resin, which reduces the likelihood of entrapment. However, two exceptions are recorded: few specimens of *Melikertes* bee and a beetle. The *Melikertes* bee was likely polylectic and resin-collecting, and may have foraged on members of Dipterocarpaceae and other plant families to gather resin for nest construction.^19^ In extant tropical rainforest, bees are the primarily pollinators of canopy trees, playing a crucial role in maintaining canopy floral biodiversity.

Beetles, in contrast to bees, are well recognized as effective pollen vectors for understory and sub-canopy vegetation, particularly among several angiosperm families, such as Araceae, Myristicaceae, and Arecaceae. In addition, the presence of a diverse array of non-biting midges in the ULM amber suggests that they, too, may have played a functional role in pollination, especially of small-flowered or wind-assisted taxa within the herbaceous and shrub layers. Given their distinct ecological roles, bees, beetles, and non-biting midges collectively contributed to the pollination of middle Eocene rainforest vegetation, bees to the canopy dominant taxa like dipterocarps, while beetles and midges likely serviced the understory and subcanopy floral communities. Their presence, albeit limited in diversity and abundance within the amber assemblage, provides valuable insights into the structure and functional dynamics of Eocene tropical rainforest pollination systems (Figure 8; Table S1).

### Eocene: A period of biodiversity hotspot on the Indian plate

The Eocene Epoch marked a peak in Indian paleobiodiversity. During this time, global warmth, combined with the Indian Plate’s progressive drift towards and beyond the equator and its quasi-isolated island character, facilitated a stable, long-term tropical climate. This setting fostered the diversification of a variety of life forms, especially as the region transitioned from a seasonal climate in the Paleocene-early Eocene to a perhumid climate in the middle Eocene. The resulting environmental conditions spurred a major diversification of faunal communities.^7^^,^^114^^,^^115^ A notable increase in biodiversity is recorded across several groups, including mammals, whales, rodents, Cambaytherians, and bats,^108^^,^^116^^,^^117^ together with fishes, ostracods, birds, frogs, and reptiles (e.g., snakes).^106^^,^^118^ Furthermore, plant families, such as Fabaceae,^119^ Arecaceae,^33^ Euphorbiaceae,^120^ and Dipterocarpaceae^22^ also experienced significant diversification during this time.^21^ These changes reflect an overall warming and humidification that supported complex tropical ecosystems.

The existence of a middle Eocene diverse arthropod biota from the ULM amber corroborates the molecular, chemical, and palynological evidence, advocating the presence of rich arborescent standing wet tropical forest vegetation along with abundant dead and decaying leaf litter and wood in the forest floor. Notably, the ULM also shares continuity with the older amber from the Cambay basin (Ypresian: Early Eocene), demonstrating common botanical origins, similar arthropod assemblages (e.g., *Melikertes* bees), and comparable depositional conditions (Table S2).^19^ As such, the fossil assemblages from the Indian Eocene amber archive what is arguably the most diverse, early Asian rainforest communities from the Paleogene, illustrating that the antiquity of tropical forests was not largely restricted to the Western Hemisphere.

Our findings align with the “stability and time” variables of the ESAT theory of tropical diversity. This theory posits that tropical taxa accumulate over tens of millions of years under relatively stable climatic conditions, with lower extinction rates compared to other environments.^5^ The long-term persistence and resilience of Indian tropical rainforests, which endured through major climatic events, such as the Deccan-induced hyperthermal event, the Paleocene-Eocene Thermal Maximum (PETM), and the Early Eocene Climatic Optimum (EECO), further reinforce this model.^7^^,^^8^^,^^18^ These forests not only survived but also diversified exponentially under the high-temperature and high-precipitation conditions of these climatic events.^7^^,^^8^ Indeed, the late Maastrichtian flora of India is considered remarkably modern and among the most diverse globally.^8^^,^^18^

Persistently, the tropical biota expanded geographically and diversified over much of the Indian continental plate during the Eocene, facilitated by the Indian plate’s favorable palaeolatitudinal position (5° S–22° N) within the equatorial humid belt.^35^^,^^121^ It is well established that larger ecosystems tend to support higher species diversity.^6^^,^^122^^,^^123^ Similarly, the middle Eocene biota from ULM included a great diversity of flora and fauna, comprising over 50 plant families and approximately 45 arthropod families across 15 orders. The quasi-isolated island nature of the Indian Plate, coupled with its vast expanse, further fostered intense biotic diversification.^9^^,^^14^ Additionally, during the northward journey of India, the subcontinent established biotic linkages with neighboring regions, particularly from the Gondwanan and Laurasian continents.^9^^,^^14^ The recovered middle Eocene flora reflects a mixed biogeographic affinity, showing contribution from both Gondwanan and Laurasian origin elements. Fossil representatives of NLRs, such as Casuarinaceae, Nothofagaceae, Proteaceae,^124^ Lythraceae,^125^ Araucariaceae, *Myrtaceae* families along with *Lagerstroemia* and *Gunnera*, indicate the persistence of Gondwanan relicts during the middle Eocene.^126^ Futhermore, palynological taxa typical of perhumid/wet forests (e.g., *Longapertites*) and seasonal dry forests (*Margocolporites*) reinforce the Gondwanan legacy of the vegetation.^126^ Conversely, the presence of fossil taxa such as *Callophyllum* and members of Euphorbiaceae and Rubiaceae^127^ suggest Laurasian affinities. Prior to final collision with Eurasia, the Indian Plate established a transient biotic connection with Africa via the Kohistan-Ladakh arch.^8^^,^^35^^,^^42^ At the same time, the African Plate maintained connections with South America through the Rio Grande Rise-Walvis Ridge system and with Laurasia via the Intra-Tethyan Island Arc system.^35^^,^^42^ These intercontinental connections facilitated the bidirectional dispersal of biota.^128^^,^^129^ India, thus, acted as a biogeographical conduit, receiving Laurasian and South American flora indirectly via African Plate, ultimately promoting a quasi-biotic exchange between Gondwanan and Laurasian realms.^128^^,^^129^ The biotic exchange, alongside the global spread of low-latitude taxa during periods of global warmth, contributed to the diversification of India’s Eocene biota.^26^ The late Paleocene and Eocene epochs were the warmest in the Cenozoic, characterized by a lack of polar or glacial ice, allowing subtropical species to thrive even at high latitudes (up to 75° paleolatitude).^130^ This global “hothouse” climate, one of the most biodiverse periods in the Phanerozoic, provided the ideal conditions for the evolution of rich tropical biota, particularly in regions, such as western and northeastern India.^29^^,^^131^ However, despite its biotically rich fossil record, India’s contribution to the extant biodiversity of the Asian tropics is somewhat limited. The tectonic and climatic shifts that occurred during India’s northward journey, coupled with the cooling of the climate in the late Eocene, led to the loss of much of the native flora.^132^ This loss was compounded by further geological and climatic changes, which caused a contraction of the tropical rainforest ecosystems to smaller regions of the Indian subcontinent by the late Eocene.^133^

The middle Eocene tropical ecosystem reconstructed in this study, characterized by a highly diverse biota, intricate trophic relationships, and specialized mutualisms, provides a valuable analog for present-day tropical forests under warming scenarios. The co-evolution of angiosperms with diverse arthropod guilds including pollinators, herbivores, fungivores, and parasitoids underscores the long-term ecological significance of mutualistic networks. These interactions, well established by the Eocene, likely contributed to the structural and functional resilience of the ecosystem despite elevated mean annual temperatures and rainfall seasonality. Drawing parallels to modern tropical systems, our findings suggest that the stability of biotic networks, rather than temperature alone, is critical for sustaining biodiversity. As contemporary climate change accelerates, these insights emphasize the urgent need to protect not only biodiversity per se but also the ecological interactions and evolutionary processes that underpin tropical ecosystem resilience and adaptability.

### Limitations of the study

Paleobotanical methods like the NLR and CA assume that fossil taxa share climatic preferences with their modern counterparts. However, evolutionary changes can lead to significant ecological and climatic divergence over time. While these methods are robust tools for paleoclimate and paleoecological reconstructions, such assumptions may introduce uncertainties and potential biases in the results.

## Resource availability

### Lead contact

Requests for further information, resources, and reagents should be directed to and will be answered by the lead contact, Shreya Mishra (shreya@bsip.res.in).

### Materials availability

All the arthropods’ specimens and permanent glass slides used for palynological studies given in all the figures (Article and supplementary figures) are deposited at the museum repository of the Birbal Sahni Institute of Palaeosciences (BSIP), Lucknow, India. The amber arthropods have been deposited with well-labeled and well–cataloged repository boxes, to prevent any mechanical damage. Permanent palynological slides have been prepared using Canada balsam to avoid any desiccation of the organic matter over time. All the slides and specimens are publicly available after gaining respective permissions. The fossil specimens were recovered from the sediments of the middle Eocene Harudi Formation of the Umarsar Lignite Mine, Kutch Basin, Gujarat, India. All necessary permits were obtained for the prescribed study, which complied with all relevant regulations. In all the years in which BSIP dug for fossils in the Umarsar Lignite Mine, it had permission to do so issued by the following authority: Gujarat Mineral Development Corporation Ltd., Kutch Basin.

### Data and code availability

A list of all fossil arthropods, pollen, biomarker, macerals identified in the studied section of Umarsar Lignite Mine, Kutch Basin, is given in the supplemental information (Table S1; Data S1 and S2). The table also includes extant distribution and paleoecological characteristics of the entire recovered biota (Table S1).

Any additional information required to reanalyze the data reported in this publication is available from the lead contact upon request.

## Acknowledgments

The authors acknowledge the Birbal Sahni Institute of Palaeosciences, Lucknow, for providing the necessary infrastructural facilities to accomplish this research (BSIP/RDCC/83/2024-25). We express our sincere gratitude to Prof. Ashok Sahni, Panjab University, Chandigarh, for his invaluable support and persistent guidance throughout this study. We also extend our thanks to the authorities of the Gujarat Mineral Development Corporation (GMDC), Gujarat for their help, support, and cooperation during the field investigation of the Umarsar Lignite Mine, Kutch.

## Author contributions

Conceptualization, S.M. and P.A.; methodology, S.M., P.A., H.S., V.P.S, K.A.S., S.D., and T.P.; software, S.M.; formal analysis, S.M., P.A., H.S., V.P.S, K.A.S., S.D., and T.P.; investigation, V.P.S., P.A., H.S., D.G., S.M., S.D., T.P., and K.A.S.; resources, V.P.S., P.A., H.S., D.G., S.M., S.D., T.P., and K.A.S.; data curation, P.A., S.M., and H.S., writing-original draft, S.M.; writing-review and editing, S.M., H.S., D.G., S.D., P.A., V.P.S., M.G.T., and K.A.S.; visualization, S.M. and P.A.; supervision, H.S., D.G., and M.G.T.; project administration, H.S., funding acquisition, H.S., D.G., and M.G.T.

## Declaration of interests

The authors declare no competing interests.

## STAR★Methods

### Key resources table

**Table:** 

REAGENT or RESOURCE	SOURCE	IDENTIFIER
**Chemicals, peptides, and recombinant proteins**		

Toluene	ThermoFisher Scientific	268370025
Acetic anhydride	ThermoFisher Scientific	4340865
Sulfuric acid	ThermoFisher Scientific	Q29995
Polyvinyl Alcohol	ThermoFisher Scientific	ALF-041241-14
Canada Balsam	ThermoFisher Scientific	C264048
Dichloromethane	ThermoFisher Scientific	113460250
Methanol	ThermoFisher Scientific	M405815
Pentane	ThermoFisher Scientific	P102415
n-hexane	ThermoFisher Scientific	ALF-047104-AK

**Deposited data**		

Arthropods specimens	Repository of Birbal Sahni Institute of Palaeosciences (BSIP), Lucknow	BSIP Specimen Nos. 42212, 42213, 42215, 42216, 42218–42220, 42223
Permanent glass slides	Repository of Birbal Sahni Institute of Palaeosciences (BSIP), Lucknow	BSIP Slide Nos. 17462–17464, 17466, 17468, 17470, 17473–17475, 17479-17483

**Software and algorithms**		

Petroglite 2.35 software	https://ws2.petrog.com/petroglite/petroglite.html	
Leica Application Suite X (LAS X)	https://www.leica-microsystems.com/products/microscope-software/p/leica-las-x-ls/	
Chem station software	https://www.agilent.com/en/product/software-informatics/analytical-software-suite/chromatography-data-systems/openlab-chemstation	
Corel Draw Graphic Suite 2021	https://www.coreldraw.com/en/product/coreldraw	
Palaeoflora Database.	http://www.palaeoflora.de	

**Other**		

JEOL JSM 7610F (SEM)	JEOL Microscopes	https://www.jeol.com/products/scientific/sem/JSM-7610F.php
Leica M205A stereoscope	Leica Microsystems	https://www.leica-microsystems.com/products/light-microscopes/stereo-microscopes/p/leica-m125-c/
Olympus BX63 Light Microscope	Olympus Microscopes	https://evidentscientific.com/en/products/upright/bx63
Leica DM4P petrological Microscope	Leica Microsystems	https://www.leica-microsystems.com/products/
Agilent 7890A Gas chromatogram	Agilent Technologies	https://www.agilent.com/
Agilent 5975C mass spectrometer	Agilent Technologies	https://www.agilent.com/

### Method details

#### Material

At present, the mine exposes four lignite seams, ranging from a few centimeters to around 2 m in thickness, the upper lignite seams are thinner than the basal seams (Figure 1C). Eight samples, including seven lignite and one dark gray shale, were collected, along with amber nodules. The palynological and entomological investigation was conducted on the amber nodules. Petrographical analyses were performed on all lignite samples, except shales; biomarker analyses were carried out only on four representative samples (Figure 1C).

#### Extraction of arthropods and pollen from amber

The Indian Eocene amber (Kutch and Cambay basins), being a Class II, cadinene-based (dammar-type) of fossil resin, gets easily dissolved, whereas Class I ambers (sesquiterpene-based, e.g., Domnician and Baltic amber) are too crosslinked for dissolution. This aspect of Indian Eocene amber uniquely allows extraction of the spores and pollen for detailed palynological analysis. Pollen and insects were extracted from the Kutch amber based on the methodology given by Rust et al.,^19^ and Singh.^78^ The procedures for arthropods and pollen extraction from amber are slightly different. For the extraction of arthropods from amber, each inclusion-containing slab was placed on aluminum foil and completely submerged within the toluene. The setup was left undisturbed until the entire solvent evaporates, leaving behind the entire organic matter containing arthropods fossils. The inclusion that remained on the foil was dried and placed on an aluminum stub, conducted with a silver dag, and coated with gold-palladium alloy. The stub was then placed under FESEM model JEOL JSM 7610F apparatus for SEM imaging. For studying arthropods within amber nodules, the methodology is entirely different. Primarily, amber inclusions are rinsed with running water to remove any superfluous particles, such as clay and sand and then ground using a flat lapidary wheel. The finest amber pieces with excellently preserved arthropods fossil were polished and imaged using a Leica M205A stereoscope. The detailed taxa list with their paleoecology and extant distribution is given in Tables S2 and S3.

For the extraction of palynomorphs, about 25–30 g of small amber nodules were taken in a beaker and dissolved using toluene. The setup was stirred intermittently for an hour, until all the nodules were fully dissolved. This dissolved mixture was then sieved using a 600-mesh sieve, washed in toluene, followed by distilled water and glacial acetic acid. The mixture was then centrifuged. An acetolysed mixture was prepared using acetic anhydride and concentrated sulfuric acid in the ratio 9:1, mixed with the sample material. It was kept over a sand bath for 5 min until the solution begins to boil. This mixture was then centrifuged again for the removal of the supernatant acetolysing mixture and the residue was later washed with distilled water. Once the mixture was ready, slides were prepared by smearing the residue, mixed with polyvinyl alcohol on the glass cover slips. The cover slips after drying were mounted on the glass slides using the Canada balsam. These slides were then observed and photographed under the high-resolution Olympus BX63 microscope. A complete list of palynomorphs taxa and their paleoecological characteristics (habit, habitat, pollination, etc.) is given in Table S1. The photographs of ecologically significant palynomorphs are given in Figure 4.

#### Nearest living relative (NLR) and coexistence analysis (CA)

The nearest living relative (NLR) method extrapolates the climatic parameters of extant to morphologically similar fossil taxa and assumes that both of them are related and share similar physiological requirements for climate.^46^^,^^47^ This approach is also used to drive information about the climate, ecology and the paleoenvironment. Depending on the state of preservation and morphological traits of the fossil taxon, the taxonomic identification level varies between family, genera and species. The list of fossil palynomorphs with their NLRs is given in Table S2. The NLR data of recovered assemblage (flora and fauna) is utilized for paleoecological, paleoenvironmental and paleoclimatic reconstructions.

To reconstruct the climate variables from the microfossil floral record we used the Coexistence Analysis (CA). The Coexistence Approach (CA) method is also a proven technique for estimating paleoclimatic parameters for palynoassemblages dating as old as the Cretaceous.^45^^,^^46^^,^^134^^,^^135^^,^^136^^,^^137^ The Coexistence approach works on the principle that the climatic tolerances of a fossil taxon are similar to those of their extant counterparts. The climatic parameters are calculated based on the overlapping range of tolerances for all the NLRs (Nearest Living Relatives) used for the analysis (Data S1). The CA approach is abundance and organ-independent, so the presence and absence data of either megafossil or microfossil can be utilized as long as their extant relatives (NLRs) are identifiable. The Paleo-Flora Database is used as a source for the climatic requirements of identified NLRs.^45^ For CA analysis, a minimum of 10 NLR taxa are required for reliable climate inferences.^46^ However, in the present study 80 NLRs have been utilized for the extraction of climatic parameters (Data S1). In this study, three temperature and four precipitation variables are reconstructed (Figure 3): mean annual temperature (MAT), coldest month temperature (CMT), warmest month temperature (WMT), mean annual precipitation (MAP), mean precipitation of dry, wet and warm months (MPdry, MPwet and MPwarm).

#### Organic petrography

The lignite pallets were prepared by embedding crushed sample fragments (∼0.8–1 mm), adhering to the specifications outlined in ISO 7404-2, 2009. The identification of various macerals was carried out following ISO-7404-3, 2009 and reflectance measurements followed the guidelines of ISO-7404-5, 2009. The nomenclature for different macerals, as postulated by the International Committee for Coal and Organic Petrology System 1994,^138^^,^^139^ was carefully observed. Maceral counting, involved 500 counts per sample, was conducted using Petroglite 2.35 software linked to the Leica DM4P petrological microscope employing a single-scan method. Reflectance measurements on ulminite (huminite) grains, involving 50 measurements per sample, were carried out using Leica Coal Expert software. The photomicrographs of different macerals and associated mineral matter were taken using the Leica Application Suite X (LAS X). The result of the organic petrographic analysis is furnished in Table S4, and photomicrographs illustrating the representative macerals are shown in Figure S3.

#### Gas chromatography-mass spectroscopy (GC-MS) analysis

Samples from the early Paleogene lignite mine of the Kutch Basin have been investigated to understand the paleofloral distribution in the western Indian subcontinent. Three lignite samples and one dark-gray shale were chosen for organic geochemical analysis. Initially, the samples were crushed and kept in ultrasonic in a solvent mixture of dichloromethane and methanol (9:1) to extract the bitumen. This extract was kept overnight in pentane to separate the asphaltene. The remaining maltenes were dried and fractionated using solvents of different polarities to recover the saturated and aromatic fractions. *n*-hexane (20 mL) was used to fractionate the saturate fraction and a mixture of *n*-hexane and dichloromethane (4:1, 40 mL) was used to obtain the aromatic fraction after passing through activated silica. Both the fractions were separately analyzed using the Agilent 7890A Gas chromatogram coupled with the Agilent 5975C mass spectrometer. DB-1 capillary column of 30 m × 0.25 mm. i.d., 0.25 μm film thickness was used inside Gas Chromatogram and helium was used as carrier gas with a flow rate of 1 mL/min. The GC oven temperature was kept constant at 40°C for 5 min and then raised to 310°C for 5.5 min with a ramp of 4 °C/min. Samples were analyzed in full scan mode with 70 eV ionization energy covering a mass range of 50–600 Da. The data were processed using chem station software and the compounds were identified based on retention time and published mass spectra. The distribution of *n*-alkanes is characterized by the partial mass chromatograms at m/z 57 from the saturate fraction (Figure S4 and Table S6). The TIC of the saturated fraction of the representative sample is shown in Figure S7 and Table S6. The various calculated biomarker parameters are listed in Table S5. The major compounds identified in the TIC of aromatic fraction are shown in Figure S8 and listed in Table S7. The distribution of *n*-alkanes is characterized by the partial mass chromatograms at m/z 191 is given in Figure S9.

## Supplemental information

Document S1. Data S1/Methods S1 and Data S2/Methods S2 (Figures S1–S10 and Tables S2–S7)

Table S1. Excel file containing additional data (complete list of palynomorphs, their NLRs, ecology, pollination mode) too large to fit in a PDF, related to Figures 4, 6–9 and supplemental Figure S1
