# Supplementary material for: Eocene amber fossils reveal how complex trophic interactions shaped tropical rainforest biodiversity
Source: iScience. 2025 Aug 25;28(9):113430. doi: 10.1016/j.isci.2025.113430 (PMC12496201; doi:10.1016/j.isci.2025.113430)
Supplement: Document S1. Data S1/Methods S1 and Data S2/Methods S2 (Figures S1–S10 and Tables S2–S7) [file mmc1.pdf]

iScience, Volume *28*

Supplemental information

Eocene amber fossils reveal how complex trophic interactions shaped tropical rainforest biodiversity

Priya Agnihotri, Vikram Partap Singh, Hukam Singh, David Grimaldi, Mahesh G. Thakkar, Tanu Priya, K.A. Subramanian, Suryendu Dutta, and Shreya Mishra

Data s1/ Methods s1: Palaeoclimate reconstruction of Middle Eocene based on 80 NLRs (Nearest Living Relatives) identified in the amber inclusions from the Umarsar Lignite Mine (ULM)

S. No.	Variables	Climatic Range	Average	Outlier
1.	MAP	2211 - 2700 mm	2455.5 mm ± 244.5	*Gordonia*
2.	MPwet	346 mm - 350 mm	348 mm ± 2	None
3.	MPwarm	206 mm - 221 mm	213.5 mm ± 7.5	*Araucaria*
4.	MPdry	36 - 43 mm	39.5 mm ± 3.5	None
5.	MAT	24.8 °C - 25.5 °C	25.15 °C ± 0.35	*Acer*, Matoniaceae
6.	CMT	24.3 °C - 25.9 °C	25.1 °C ± 0.8	*Acer, Araucaria, Gordonia,* Matoniaceae
7.	WMT	28.1 °C - 28.9 °C	28.5 °C ± 0.4	None
8.	WET/DRY	8.8	-	-

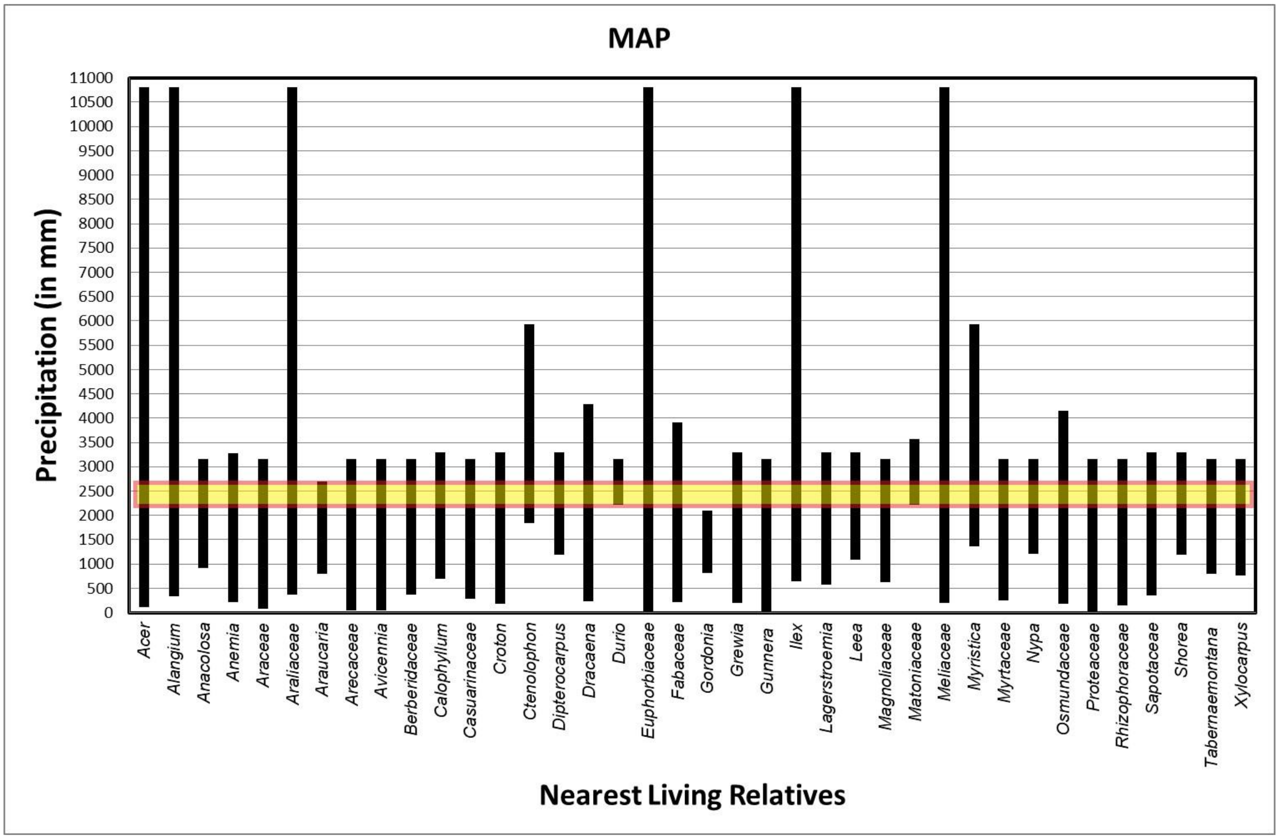
MAP
Precipitation (in mm)
Nearest Living Relatives
Acer
Alangium
Anacolosa
Anemia
Araceae
Araliaceae
Araucaria
Arecaceae
Avicennia
Berberidaceae
Calophyllum
Casuarinaceae
Croton
Ctenolophon
Dipterocarpus
Dracaena
Durio
Euphorbiaceae
Fabaceae
Gordonia
Grewia
Gunnera
Ilex
Lagerstroemia
Leea
Magnoliaceae
Matoniaceae
Meliaceae
Myristica
Myrtaceae
Nypa
Osmundaceae
Proteaceae
Rhizophoraceae
Sapotaceae
Shorea
Tabernaemontana
Xylocarpus

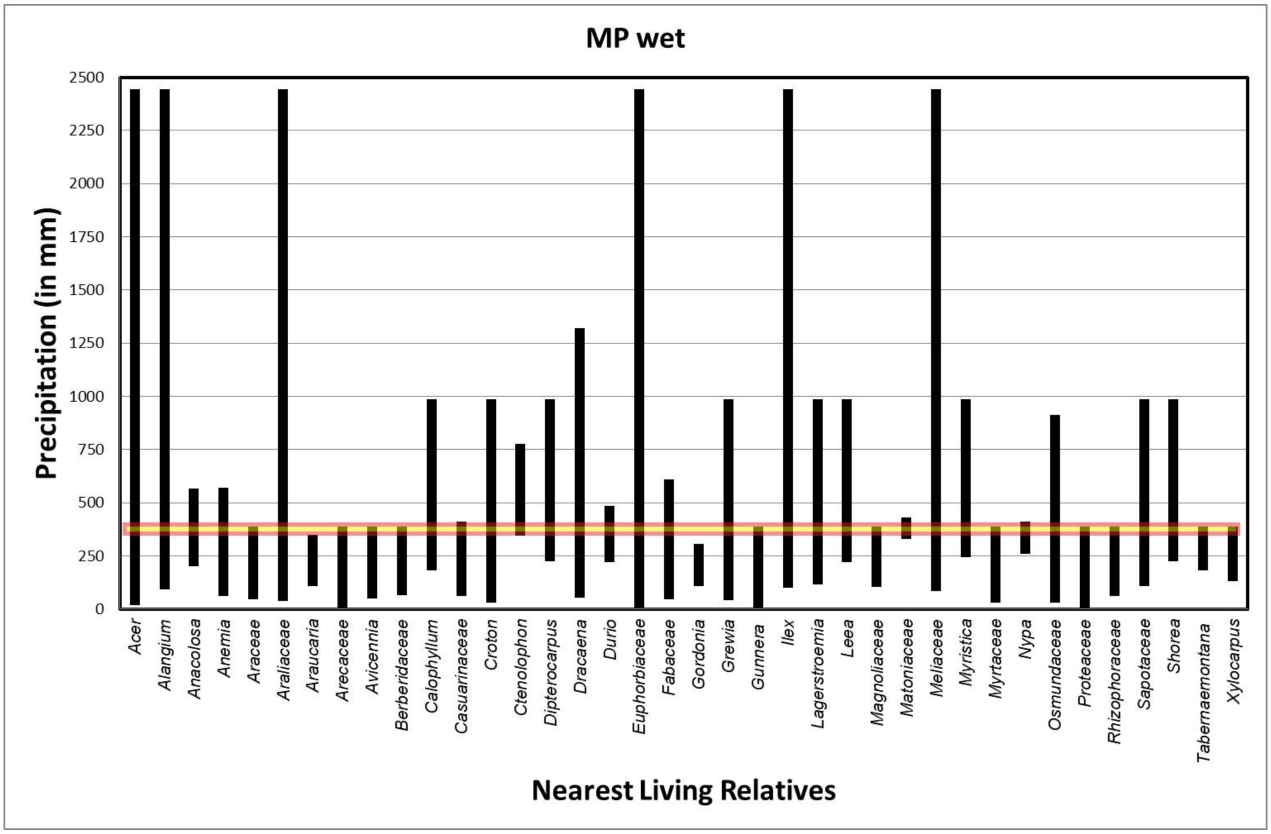
MP wet
Precipitation (in mm)
Nearest Living Relatives
Acer
Alangium
Anacolosa
Anemia
Araceae
Araliaceae
Araucaria
Arecaceae
Avicennia
Berberidaceae
Calophyllum
Casuarinaceae
Croton
Ctenolophon
Dipterocarpus
Dracaena
Durio
Euphorbiaceae
Fabaceae
Gordonia
Grewia
Gunnera
Ilex
Lagerstroemia
Leea
Magnoliaceae
Matoniaceae
Meliaceae
Myristica
Myrtaceae
Nypa
Osmundaceae
Proteaceae
Rhizophoraceae
Sapotaceae
Shorea
Tabernaemontana
Xylocarpus

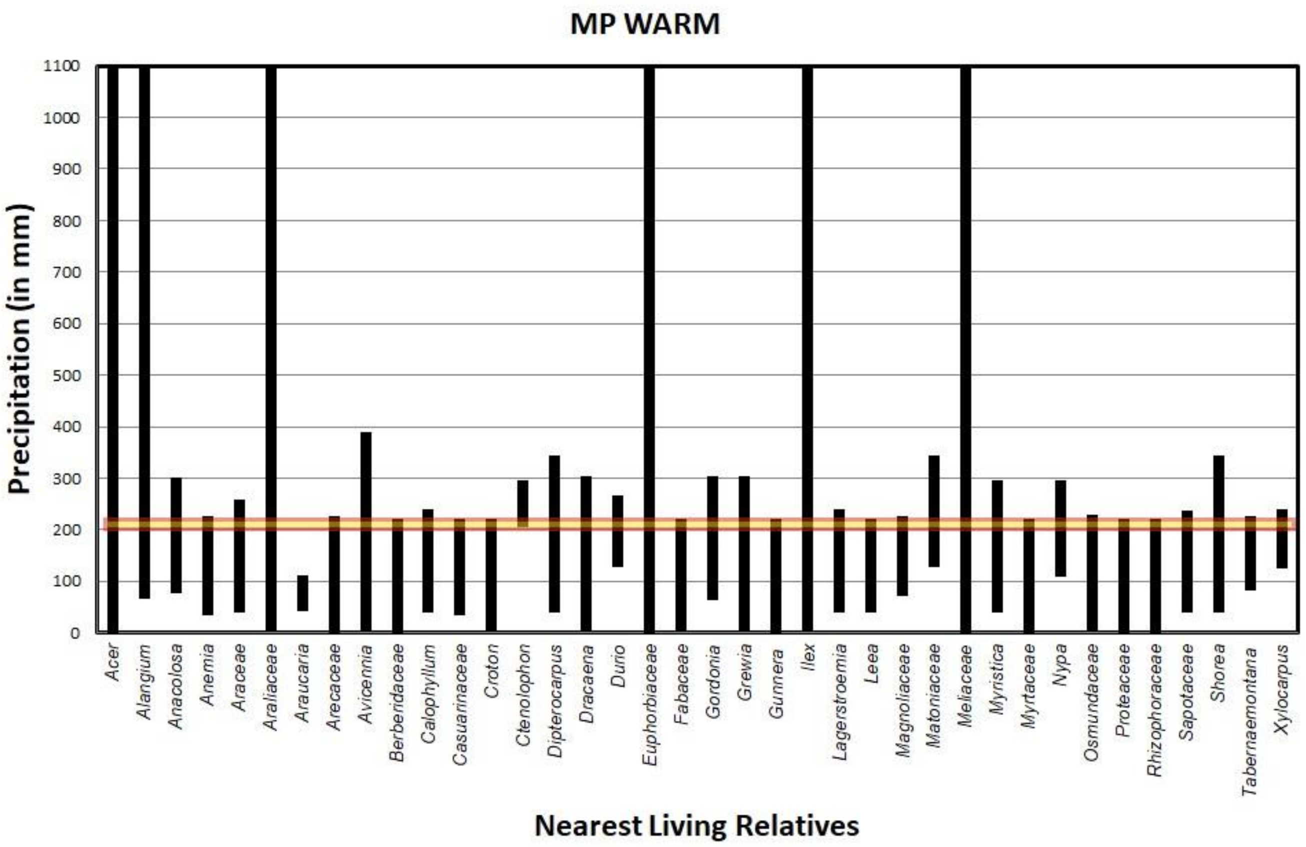
MP WARM
Precipitation (in mm)
Nearest Living Relatives
0
100
200
300
400
500
600
700
800
900
1000
1100
Acer
Alangium
Anacolosa
Anemia
Araceae
Araliaceae
Araucaria
Arecaceae
Avicennia
Berberidaceae
Calophyllum
Casuarinaceae
Croton
Ctenolophon
Dipterocarpus
Dracaena
Durio
Euphorbiaceae
Fabaceae
Gordonia
Grewia
Gunnera
Ilex
Lagerstroemia
Leea
Magnoliaceae
Matoniaceae
Meliaceae
Myristica
Myrtaceae
Nypa
Osmundaceae
Proteaceae
Rhizophoraceae
Sapotaceae
Shorea
Tabernaemontana
Xylocarpus

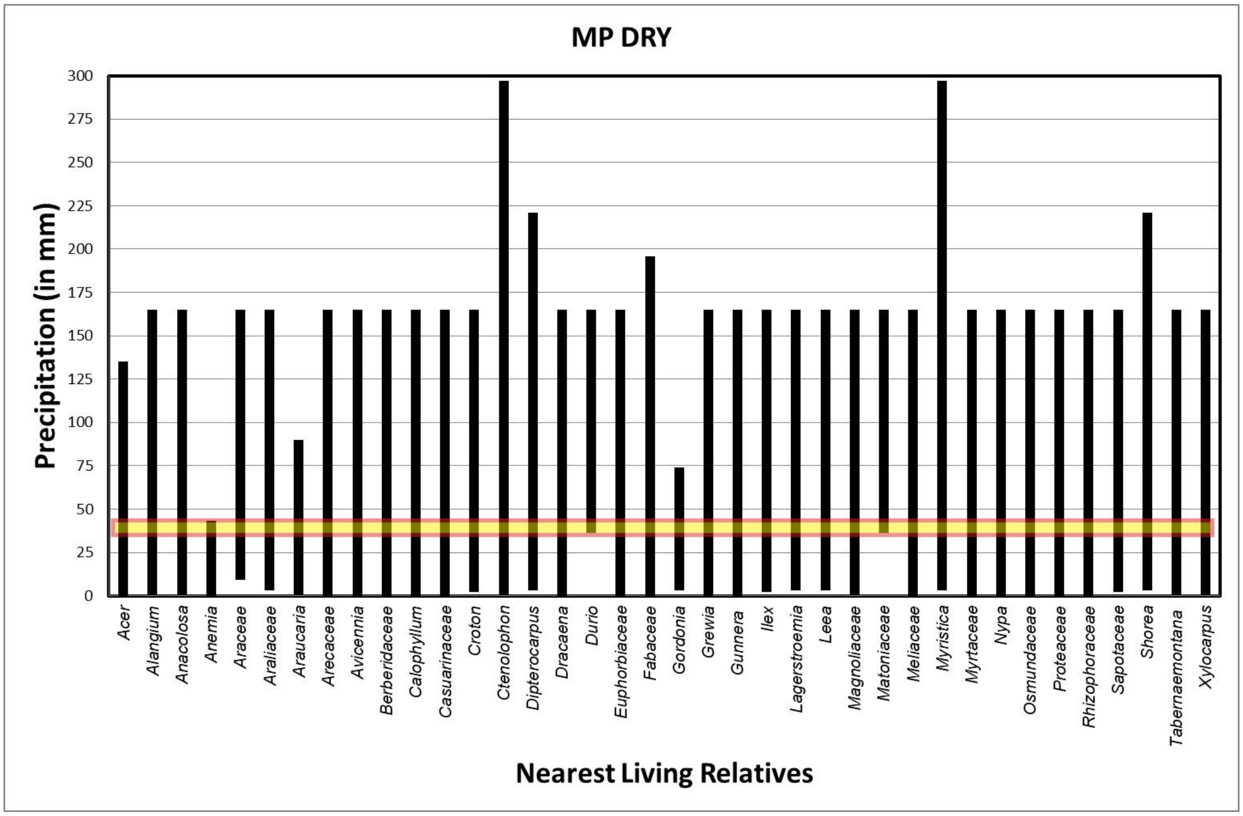
MP DRY
Precipitation (in mm)
Nearest Living Relatives
0
25
50
75
100
125
150
175
200
225
250
275
300
Acer
Alangium
Anacolosa
Anemia
Araceae
Araliaceae
Araucaria
Arecaceae
Avicennia
Berberidaceae
Calophyllum
Casuarinaceae
Croton
Ctenolophon
Dipterocarpus
Dracaena
Durio
Euphorbiaceae
Fabaceae
Gordonia
Grewia
Gunnera
Ilex
Lagerstroemia
Leea
Magnoliaceae
Matoniaceae
Meliaceae
Myristica
Myrtaceae
Nypa
Osmundaceae
Proteaceae
Rhizophoraceae
Sapotaceae
Shorea
Tabernaemontana
Xylocarpus

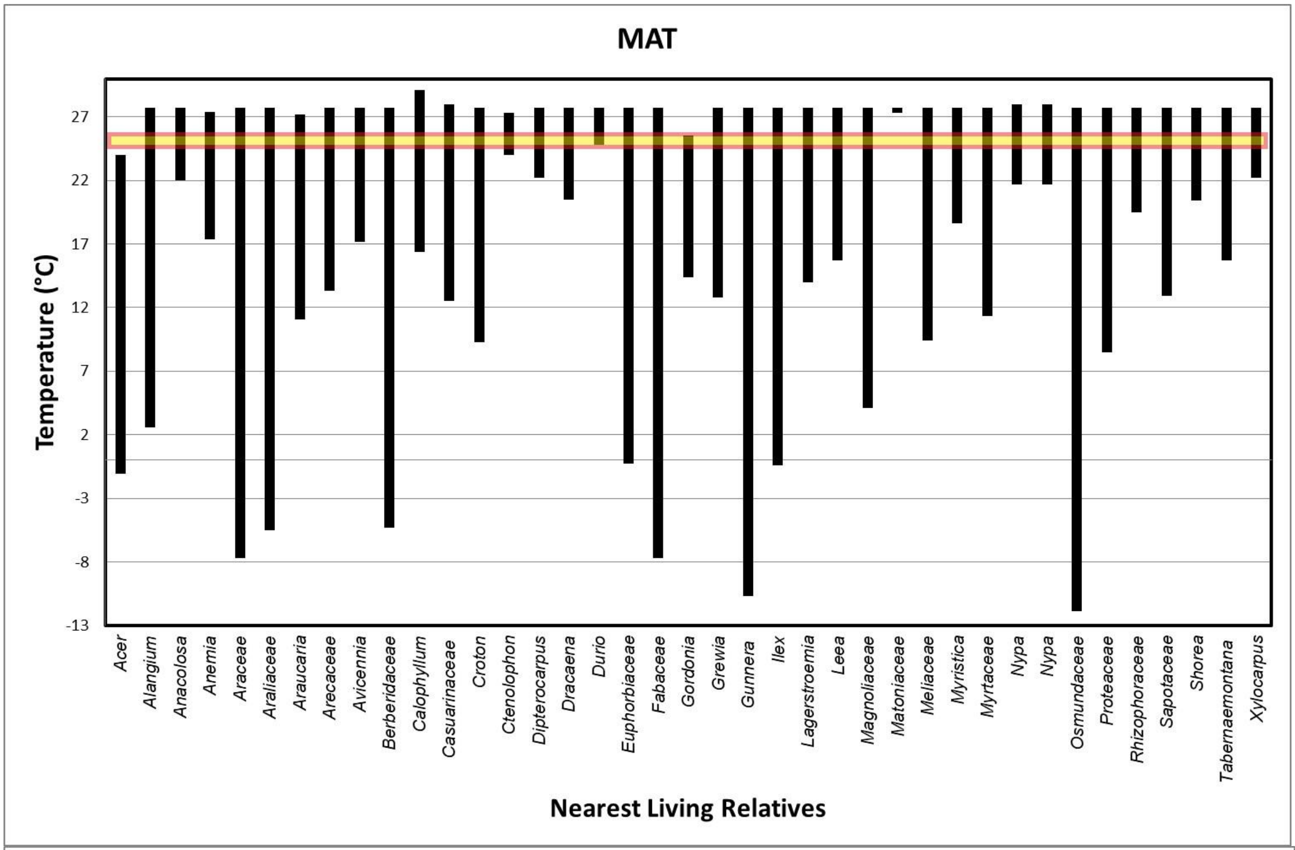
MAT
Temperature (°C)
27
22
17
12
7
2
-3
-8
-13
Acer
Alangium
Anacolosa
Anemia
Araceae
Araliaceae
Araucaria
Arecaceae
Avicennia
Berberidaceae
Calophyllum
Casuarinaceae
Croton
Ctenolophon
Dipterocarpus
Dracaena
Durio
Euphorbiaceae
Fabaceae
Gordonia
Grewia
Gunnera
Ilex
Lagerstroemia
Leea
Magnoliaceae
Matoniaceae
Meliaceae
Myristica
Myrtaceae
Nypa
Nypa
Osmundaceae
Proteaceae
Rhizophoraceae
Sapotaceae
Shorea
Tabernaemontana
Xylocarpus
Nearest Living Relatives

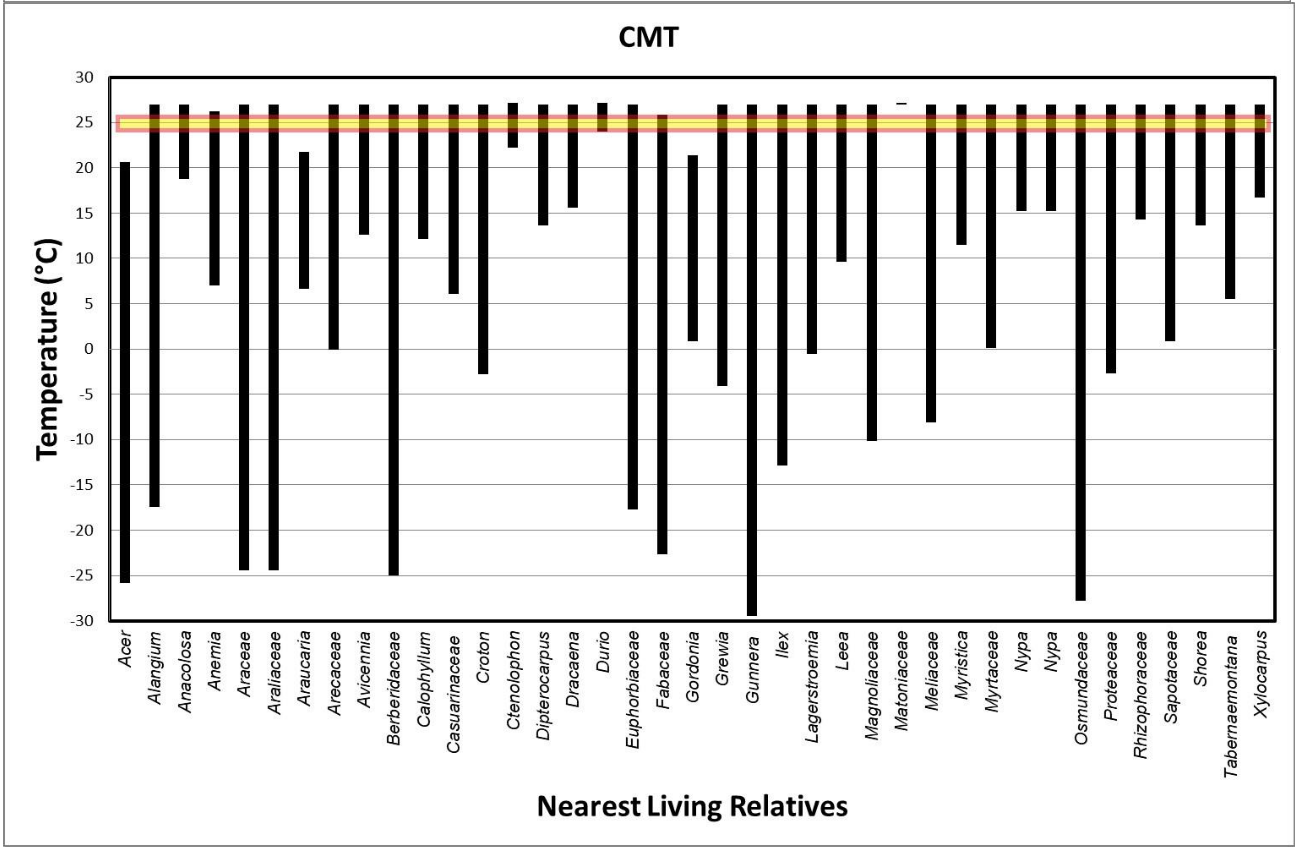
CMT
Temperature (°C)
30
25
20
15
10
5
0
-5
-10
-15
-20
-25
-30
Acer
Alangium
Anacolosa
Anemia
Araceae
Araliaceae
Araucaria
Arecaceae
Avicennia
Berberidaceae
Calophyllum
Casuarinaceae
Croton
Ctenolophon
Dipterocarpus
Dracaena
Durio
Euphorbiaceae
Fabaceae
Gordonia
Grewia
Gunnera
Ilex
Lagerstroemia
Leea
Magnoliaceae
Matoniaceae
Meliaceae
Myristica
Myrtaceae
Nypa
Nypa
Osmundaceae
Proteaceae
Rhizophoraceae
Sapotaceae
Shorea
Tabernaemontana
Xylocarpus
Nearest Living Relatives

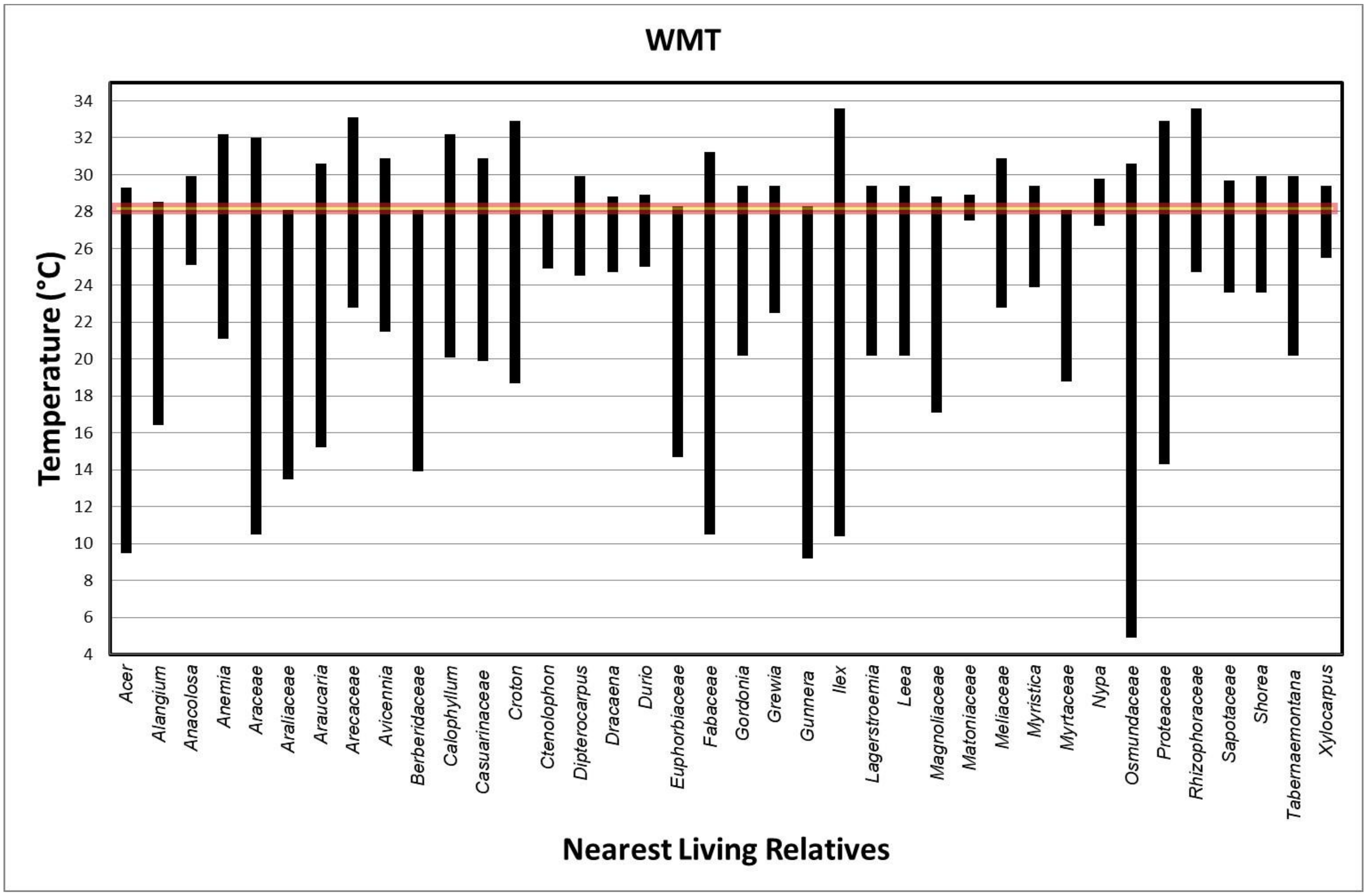

WMT
Temperature (°C)
34
32
30
28
26
24
22
20
18
16
14
12
10
8
6
4
Acer
Alangium
Anacolosa
Anemia
Araceae
Araliaceae
Araucaria
Arecaceae
Avicennia
Berberidaceae
Calophyllum
Casuarinaceae
Croton
Ctenolophon
Dipterocarpus
Dracaena
Durio
Euphorbiaceae
Fabaceae
Gordonia
Grewia
Gunnera
Ilex
Lagerstroemia
Leea
Magnoliaceae
Matoniaceae
Meliaceae
Myristica
Myrtaceae
Nypa
Osmundaceae
Proteaceae
Rhizophoraceae
Sapotaceae
Shorea
Tabernaemontana
Xylocarpus
Nearest Living Relatives

Data s2/ Methods s2: Figures S1 - S10 and Tables S2 – S7

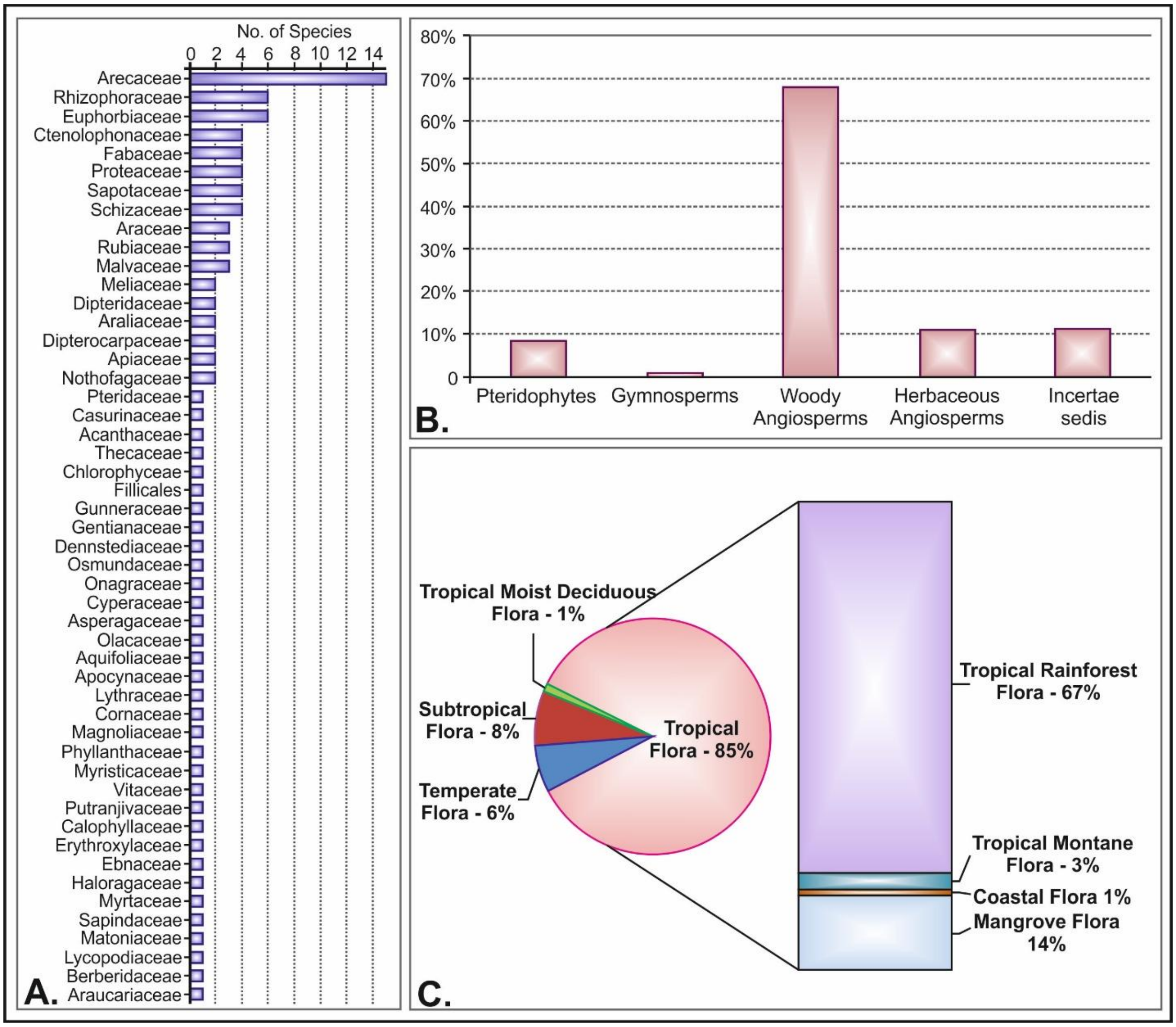

Figure S1: Taxonomic and ecological diversity of the studied assemblage. A. Taxonomic diversity of the palynomorphs recovered from the amber inclusions of Umarsar Lignite Mine relating to Table S1, B. Frequency distribution of various habits of plants relating to Table S1, C. Paleoecological preferences of the palynomorph assemblage recovered from the Umarsar Lignite Mine. Relating to Table S1.

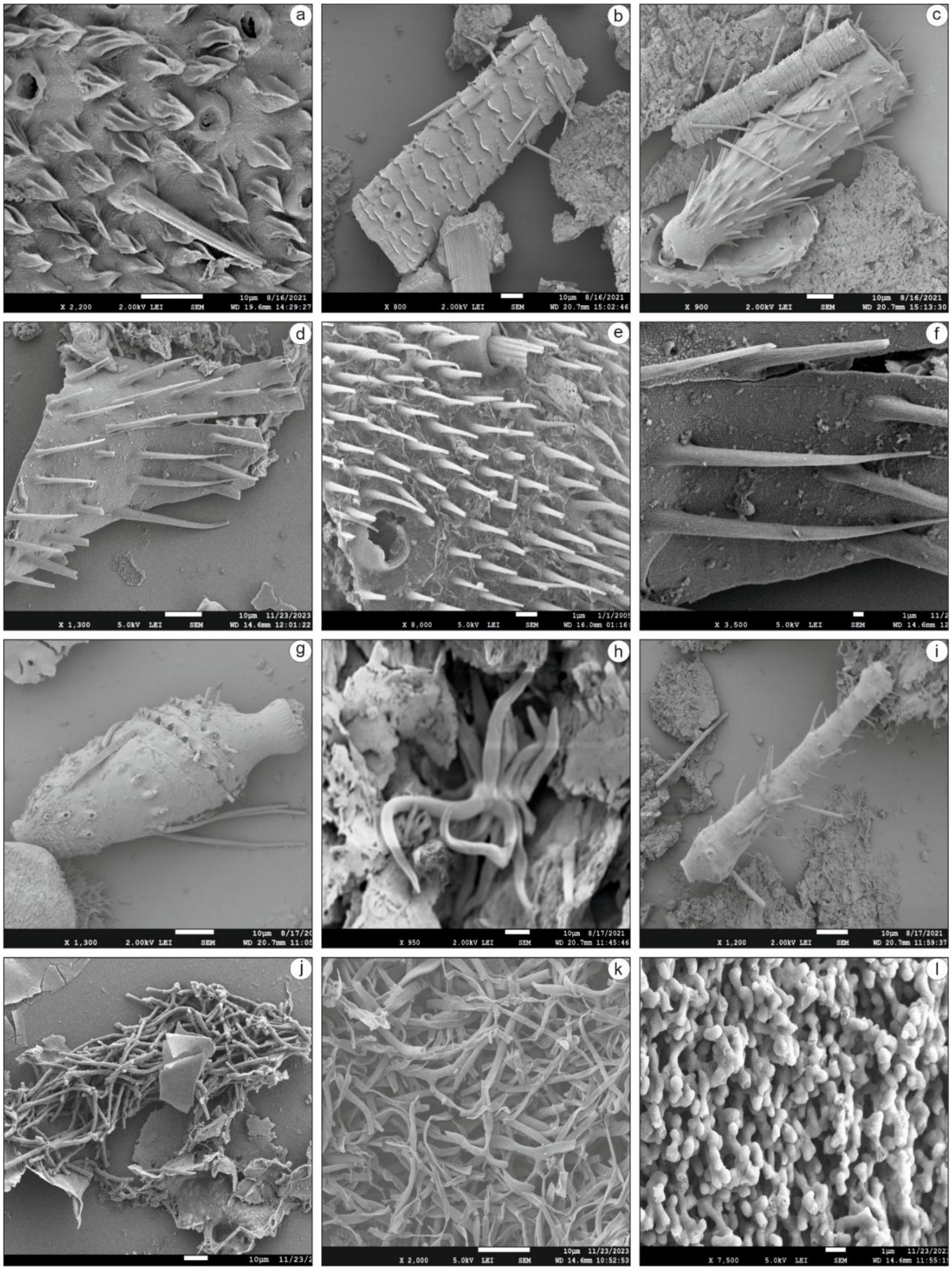

Figure S2 SEM images of amber fossils extracted from the amber. (a,e,f) insect fragments with stellate microtrichia; (b,c,d) insect cuticle with imbrications and setae; (g) antennal segment of Thysanoptra; (h) trichome segment; (i) arachnid segment (unidentified); (j,k,l) floral debris (unidentified).

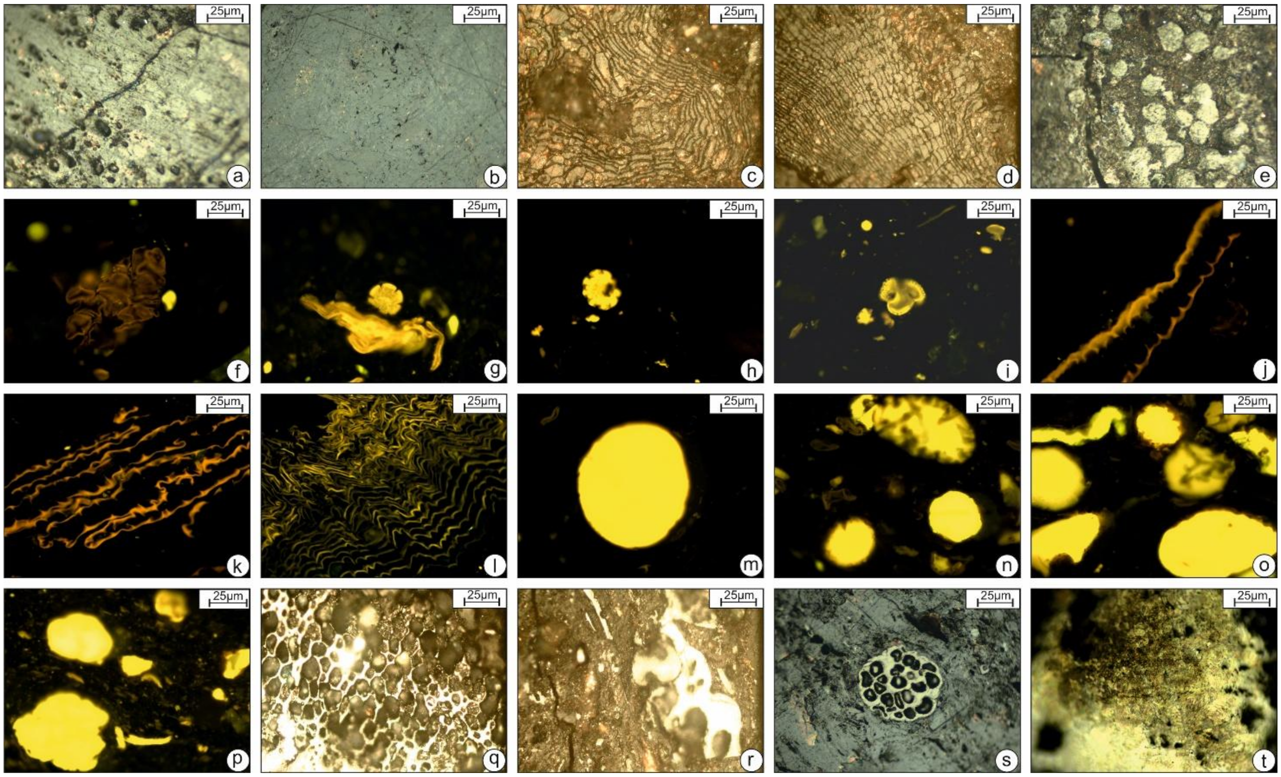

Figure S3: Representative photomicrographs of the various macerals and mineral matter recorded in the studied lignite samples. a-b) ulminite, c-d) textinite, e) corpohuminite, f-i) sporinite, j-k) cutinite, l) suberinite, m-p) resinite, q) semifusinite, r) inertodetrinite, s) funginite, t) pyrite (massive). Photomicrographs a-e and q-t are taken in incident white light while f-p are in blue light excitation (fluorescence) mode. Relating to Table S4.

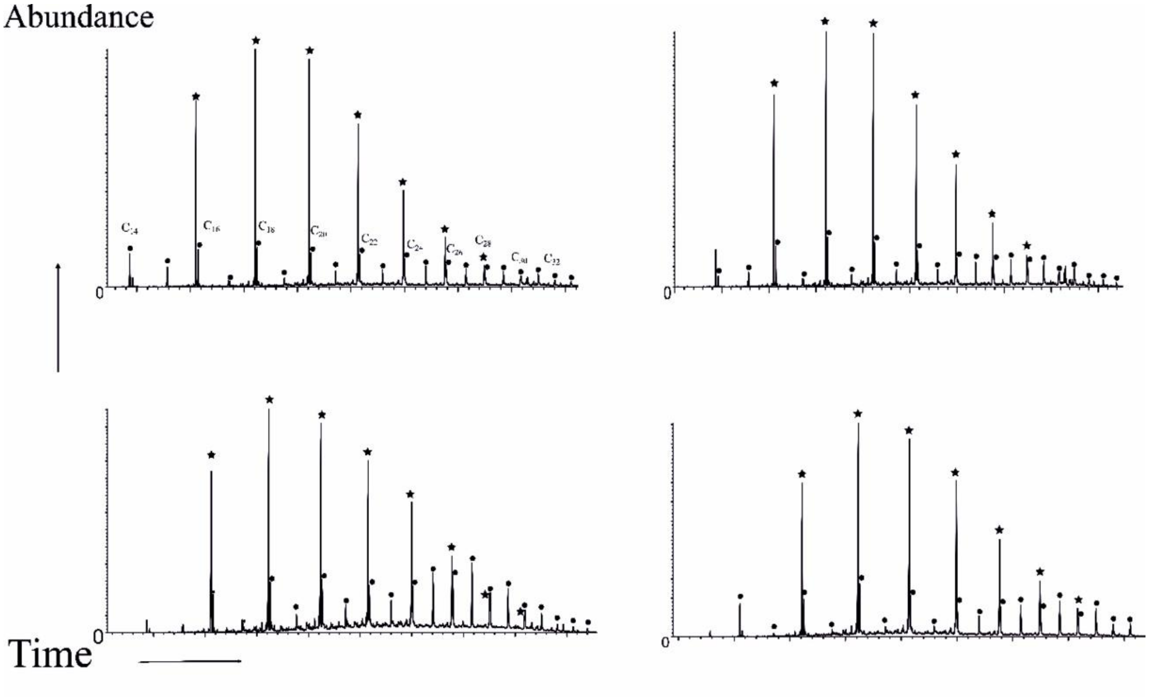

Figure S4: Partial mass chromatogram at m/z 57 of aliphatic fraction showing the distribution of n-alkanes of representative Eocene Lignite and shale samples.

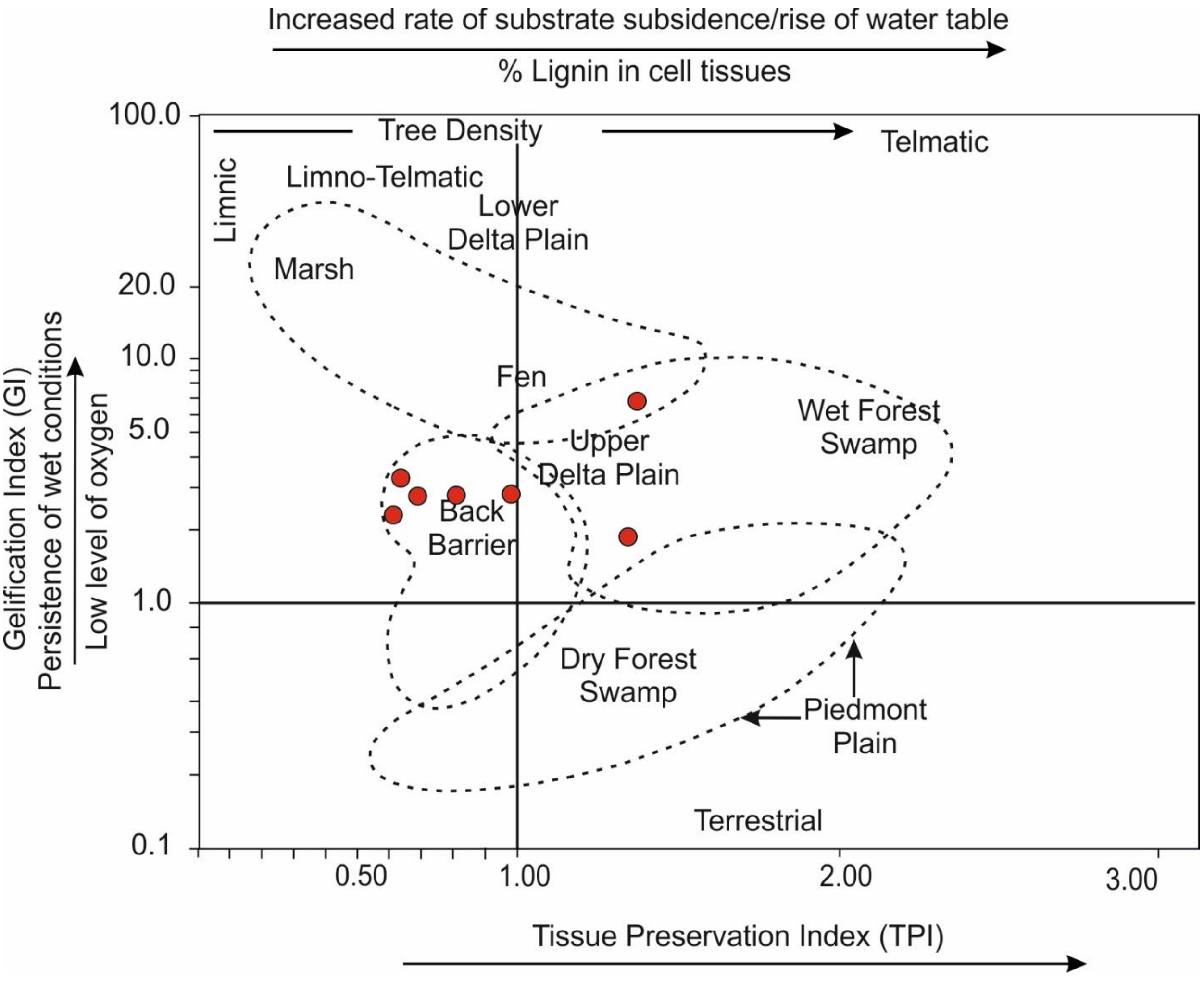

Figure S5: Facies diagram derived from the GI and TPI relationship, illustrating the depositional setting and different mire types. [S1] **Relating to Table S4.**

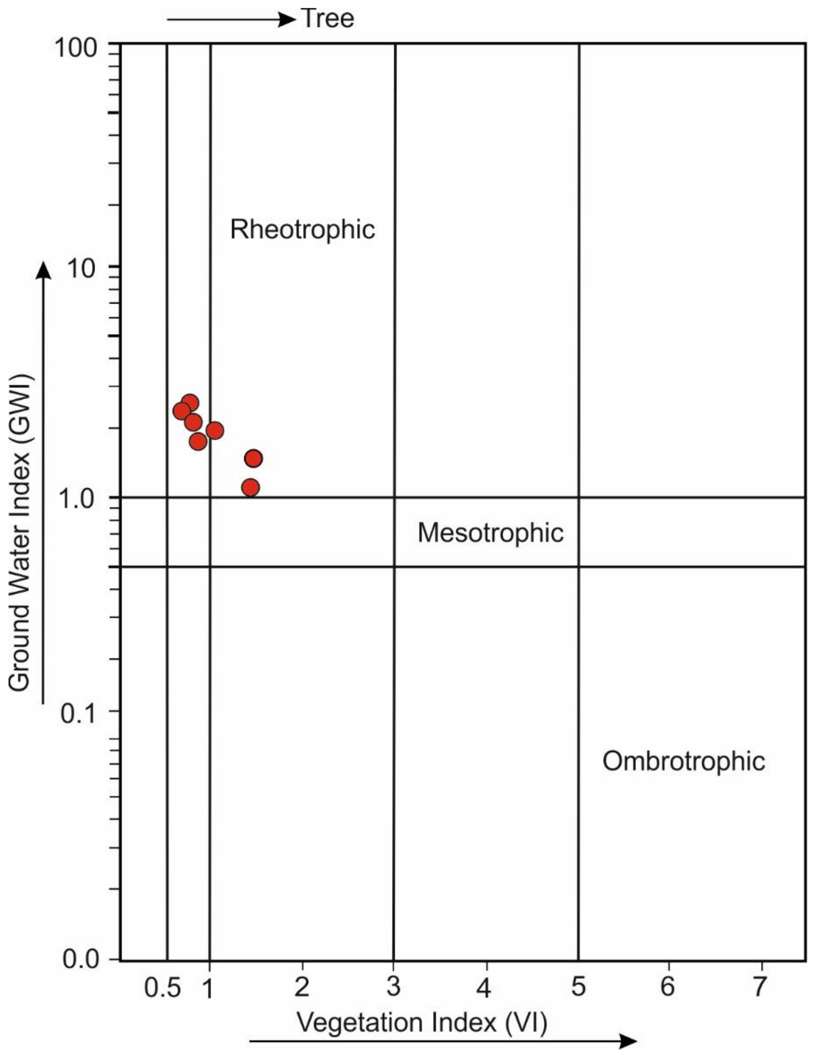

Figure S6: The VI-GWI indices diagram of the studied samples illustrates the relationship between vegetation type and the hydrological settings. [S2] **Relating to Table S4.**

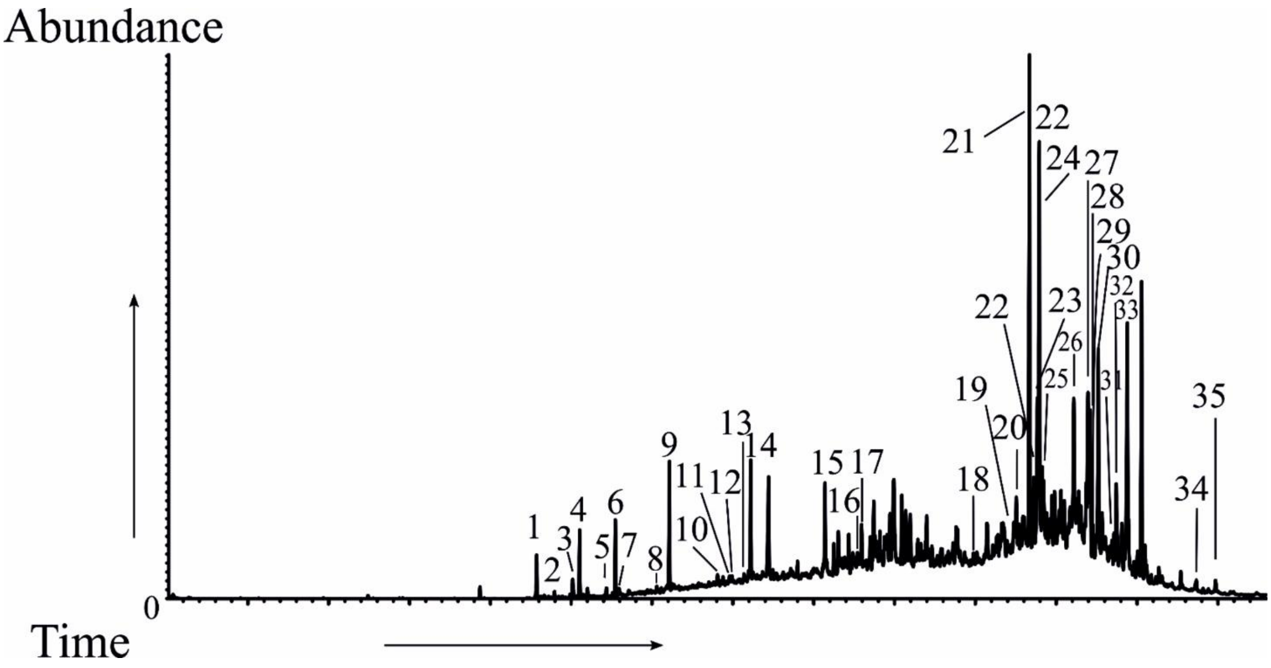

Figure S7: Total ion chromatogram (TIC) of saturate fraction of the representative middle Eocene lignite sample from Umarsar Lignite mine, Western India. Relating to Table S6.

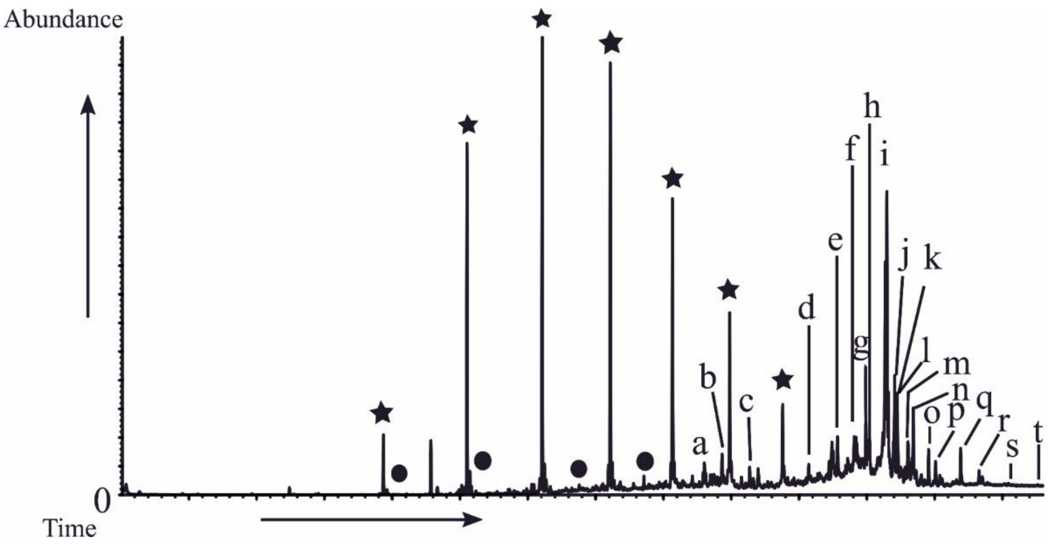

Figure S8 Total ion chromatogram (TIC) of aromatic fraction for representative middle Eocene Lignite sample from Umarsar lignite mine, Western India. Relating to Table S7.

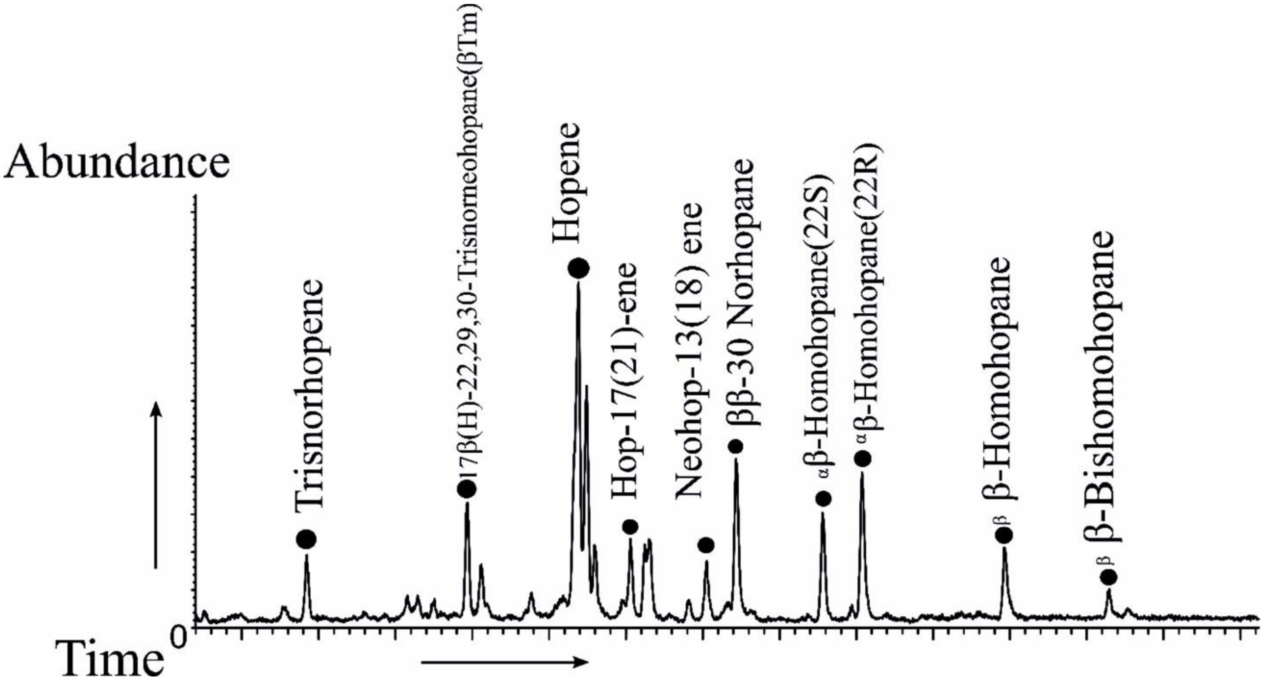

Figure S9: Partial mass chromatograms at m/z 191 for the representative middle Eocene lignite sample from Umarsar lignite mine, Western India.

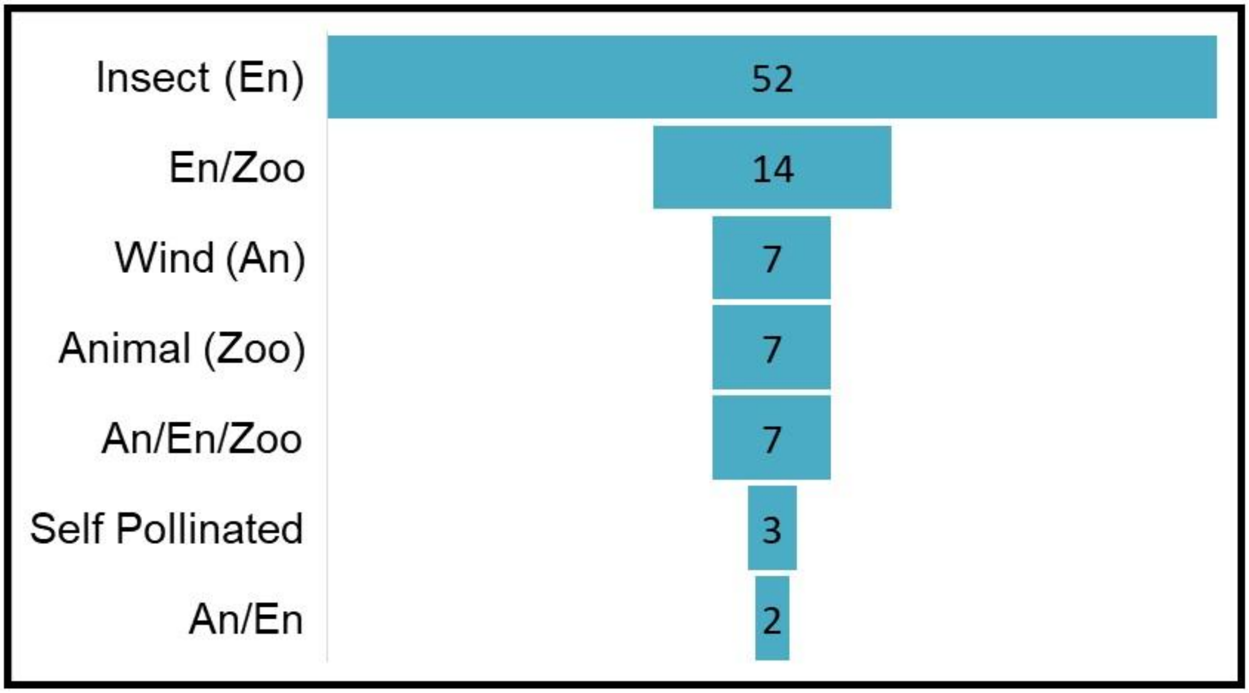

Figure S10: Different types of pollination found in the Middle Eocene tropical rainforest. Relating to Table S1

Taxon	Food habit	Umarsar Lignite Mine	Valia Lignite Mine	Tarkeshwar Lignite Mine
**ARACHNIDA**, indet.				
Araneae	Predatory/ parasitoid on terrestrial arthropods	58 (7.0%)	34 (11.7%)	61 (6.5%)
**ACARI**	Predatory/ parasitoid on terrestrial arthropods	7 (0.8%)	5 (1.7%)	86 (9.1%)
Pseudoscorpionida	Predatory/ parasitoid on terrestrial arthropods	2	1	3
Amblypygi	Predatory/ parasitoid on terrestrial arthropods	-	-	1
Scorpionida	Predatory/ parasitoid on terrestrial arthropods	-	-	1
Opilionida	Predatory/ parasitoid on terrestrial arthropods	-	-	1
Isopoda	Scavengers, saprophages, microbial/ fungal grazers	-	1	-
**MYRIAPODA**				
CHILOPODA	Predatory/ parasitoid on terrestrial arthropods	-	-	1
Millipedes	Scavengers, saprophages, microbial/fungal grazers	3	-	5
**HEXAPODA** indet.		102	28	21
COLLEMBOLA	Scavengers, saprophages, microbial/fungal grazers	(0.7%)	2(0.1%)	55 (5.8%)
Sminthuridae	Scavengers, saprophages, microbial/fungal grazers	2	-	1
Isotomidae	Scavengers, saprophages, microbial/fungal grazers	4	-	-
Entomobryidae	Scavengers, saprophages, microbial/fungal grazers		1	1
Archaeognatha	Scavengers, saprophages, microbial/fungal grazers	3	-	1
Zygentoma	Scavengers, saprophages, microbial/fungal grazers	-	-	2
Ephemeroptera	Aquatic in one or more stages	-	-	2
Odonata	Predatory/ parasitoid on terrestrial arthropods	-	-	1
Orthoptera	Phytophagous	1	5	6
Dermaptera		1	-	-
Embiodea	Scavengers, saprophages, microbial/fungal grazers	-	-	2
Blattodea	Scavengers, saprophages, microbial/fungal grazers	3	9	10
Mantodea	Predatory/ parasitoid on	-	-	5

	terrestrial arthropods			
**I**SOPTERA indet.	Scavengers, saprophages, microbial/fungal grazers	13	-	18
Termitidae	Scavengers, saprophages, microbial/fungal grazers	1	-	-
Kalotermitidae	Scavengers, saprophages, microbial/fungal grazers	1	-	-
Rhinotermitidae	Scavengers, saprophages, microbial/fungal grazers	-	-	-
Stylotermitidae	Scavengers, saprophages, microbial/fungal grazers	-	-	-
Psocodea, indet	Scavengers, saprophages, microbial/fungal grazers	36	10	19
Lepidopsocidae	Scavengers, saprophages, microbial/fungal grazers	10	-	6
**Psocodea total**		**5.5%**	**3.4%**	**2.6%**
Thysanoptera	Phytophagous	-	1	4
Hemiptera indet	Phytophagous	1	-	2
Auchenorrhyncha	Phytophagous	18 (2.2%)	10 (3.4%)	36 (3.8%)
Heteroptera	Phytophagous	8 (0.9%)	12 (4.1%)	38 (4.0%)
Miridae	Phytophagous	2	-	-
Dipsocoridae	Predatory/ parasitoid on terrestrial arthropods	1	-	-
Coccoidea	Phytophagous	2	1	1
Sternorrhyncha indet	Phytophagous	-	-	1
Trichoptera	Aquatic in one or more stages	1	-	-
Lepidoptera	Phytophagous	-	1	9
Coleoptera indet		20	21	46
Aderidae	Scavengers, saprophages, microbial /fungal grazers	1		
Dermestidae	Scavengers, saprophages, microbial/ fungal grazers	1		
Curculionidae	Phytophagous	1	-	1
Alticinae (Chrysomel.).	Phytophagous	1	12	-
Staphylinidae	Predatory/ parasitoid on terrestrial arthropods	1	3	5
Ptiliidae	Scavengers, saprophages, microbial/fungal grazers	1	-	1
Scydmaenidae	Predatory/ parasitoid on terrestrial arthropods	-	-	3
Elateroidea	Scavengers, saprophages, microbial/fungal grazers	-	-	3
**Coleoptera Total**		**2.6%**	**8.6 %**	**7.4%**
Neuroptera				
Coniopterygidae	Predatory/parasitoid on	-	-	2

	terrestrial arthropods			
Diptera indet			1	1
Brachycera indet.		1	4	10
Culicomorpha indet		97	13	116
Chironomidea	Aquatic in one or more stages	176	20	36
Ceratopogonidea	Predatory/parasitoid on terrestrial arthropods	18	7	13
Culicoidea	Aquatic in one or more stages	-	-	5
**Culicomorpha Total**		**35.3%**	**13.7%**	**18.1%**
Anisopodidae		-	-	1
Mycetophilidae		7	3	12
Sciaridae		1	0	1
Cecidomyidae	Scavengers, saprophages, microbial/fungal grazers / phytophagous	16	12	14
Scatopsidae	Scavengers, saprophages, microbial/fungal grazers	1	-	4
Psychodidae	Scavengers, saprophages, microbial/fungal grazers	7	2	6
Rhagionidae	Predatory/parasitoid on terrestrial arthropods	-	-	1
Dolichopodidae	Predatory/parasitoid on terrestrial arthropods	9	5	31
Phoridae	Scavengers, saprophages, microbial/fungal grazers	11	4	11
Tipuloidea		4	1	3
**Diptera total**		**42%**	**24%**	**28%**
Hymneoptera, indet.		11	6	2
Parasitica indet.	Predatory/parasitoid on terrestrial arthropods	6	-	23
Chalcidoidea	Predatory/parasitoid on terrestrial arthropods	4	-	9
Scelionidae	Predatory/parasitoid on terrestrial arthropods	1	6	4
Braconidae	Predatory/parasitoid on terrestrial arthropods	1	-	4
Evanioidea	Predatory/parasitoid on terrestrial arthropods	2	-	-
Bethylidae	Predatory/parasitoid on terrestrial arthropods	2	-	1
Chrysidoidea indet.	Predatory/parasitoid on terrestrial arthropods	2	-	-
Aculeata indet	Predatory/parasitoid on terrestrial arthropods	1	-	1
Formicidae Male	Predatory/parasitoid on	85	9	31

	terrestrial arthropods			
Formicidae Worker	Predatory/parasitoid on terrestrial arthropods	51	30	106
Formicidae Gynes	Predatory/parasitoid on terrestrial arthropods	5	-	3
**Ants total**		**17%**	**13%**	**15%**
Bees		5	1	4
**Total Arthropods**		**824**	**290**	**936**
**Total Arthropods in all the mines**		**2050**		

Table S2: Comparison of fossil arthropods assemblages from the different western Indian mines.

Studied Arthropods	Family	Food	Habitat preference in accordance with insect-plant interaction
**Non-biting Midges**			
Chironomus Meigen	Chironomidae Newman	Non-biting Midges Feed on dead leaves, fresh fly droppings, nectar, pollen and other sugary materials	Although eurytopic, larvae aquatic and prefer relatively clean water with high nutrient /low oxygen conditions. They are the most abundant benthic organisms. These insects are found in all sorts of environments from marine to terrestrial including rivers, streams, lakes, salt marshes, intertidal pools etc.
Tanypus Meigen	Chironomidae Newman	Non-biting Midges Feed on dead leaves, fresh fly droppings, nectar, pollen and other sugary materials	Larvae aquatic to semiaquatic, adults near water habitats associated with fresh and brackish waters. They are tolerant to high temperatures.
Lauterborniella Thienemann and Bause	Chironomidae Newman	Particles of algae and detritus in the vicinity	Aquatic to semi-aquatic; suitable near moist habitats.
Orthocladius Van der Wulp	Orthocladiinae Lenz	Feed on diatoms and fine detritus organic matter	Larvae live in flowing water, like rivers and streams
Parochlus Enderlein	Podonominae Kieffer	Particles of algae and detritus in the vicinity	Larvae and pupae are aquatic, inhabiting lakes and streams, while winged adults are terrestrial and found along the edges of lakes and streams
**Biting Midge**			
Genus indet.	Ceratopogonidae Newman	Feed on fungi, algae; adult Females feed on the blood of vertebrates, few species parasitic on other insects	Aquatic and Semi-aquatic to terrestrial habitats.
**Gall Midges**			
Ledomyia Kieffer	Cecidomyiidae Newman	Feed on fungus and plant tissue	Prefer larger plants than small ones; interaction with flower families Apiaceae, Moraceae, Piperaceae and ascomycetes
Electroxylomyia Nel and Prokop	Cecidomyiidae Newman	Feed on fungus and plant tissue	Prefer larger plants than small ones; interation with flower families Apiaceae, Moraceae, Piperaceae and ascomycetes
**Ants**			
Dolichoderus Lund	Dolichoderinae Forel	Nectar and phloem sap of young plant parts	Grows in forested areas ranging from dry savanna woodlands to rain forests.
Formica Linnaeus	Formicidae Latreille	Feed on nectar and honeydew	Ubiquitous, habitats ranging from sparse to medium dense mature forest, bogs and wetlands.
Myrmicinae indent.	Formicidae Latreille	Feed on symbiotic fungus	Ubiquitous, habitats ranging from sparse to medium dense mature forest, bogs and wetlands.
Gesomyrmex Mayr	Formicidae Latreille	Chew on heartwood and feed on honey dew	Inhabitants of tropical rain forests
**Beetle**			

Genus indet.	Chrysomelidae Latreille	Feed on variety of plant parts like, stem, leaf, flower, pollen etc.	Dominantly found in tropics and occupy sub-canopy and understory layers of the forest and prefer to eat shrubs and small trees.
**Termite**			
Genus indet.	Termitidae Latreille	Feed on materials of vegetative origin	Inhabits trees with high cellulose, including Malvaceae, Musaceae and Cannabaceae
**Mite**			
Sarcoptes Linnaeus	Sarcoptidae Linnaeus	Parasitic; few feeds on forest litter habitats including soil moss, lichens, barks and leave	Colonized terrestrial land but later evolved to parasitic mode of habitats
**Spider**			
Genus indet.	Araneidae Clerck	Prey on beetles, flies and also prey small birds, moths using web traps.	Inhabit diverse areas in the tropical rain forest ranging from forest floor to tree canopies.
**Long-legged Flies**			
Genus indet.	Dolichopodidae Latreille	Adults and larvae predate on small invertebrates, many serve as kleptoparasites on other arthropods including spiders.	Larvae are adapted to moist places like soils, moist sand and rotting vegetal matter. Adult's adapted to shrubby regions near wet biotopes.
**Mosquito**			
Mansonia Blanchard	Culicidae Meigen	Commonly blood Feeders; feed mostly on vertebrates including wild mammals.	Neotoropical mosquito whose larvae breed in water habitats while adults inhabit terrestrial grasslands.
**Ostracod**			
Candona, Limnocytheridae (unindet.) & Cytheridae (unindet.)	Candonidae Kaufmann	Freshwater detritus a diatom	Freshwater species living in warm and humid environment

Table S3: List of arthropod taxa recovered from the amber of Umarsar Lignite Mine, with their preferred food and habitats.

Sample No.	U-1	U-2	U-3	U-4	U-5	U-6	U-7	U-8	Avg.
Macerals									
**Huminite (H)**	**60.2**	**58.2**	**54.4**	**62.7**	**64.1**	**58.0**	**60.4**	**62.3**	**60.0**
**Telohuminite**	**22.1**	**31.0**	**27.2**	**16.5**	**21.1**	**21.6**	**18.9**	**18.3**	**22.1**
Textinite	0.8	0.0	0.8	0.7	0.4	1.6	1.3	2.8	**1.1**
Ulminite	21.3	31.0	26.4	15.8	20.7	20.0	17.6	15.5	**21.0**
**Detrohuminite**	**30.9**	**23.4**	**20.4**	**36.3**	**33.2**	**28.2**	**35.6**	**37.7**	**30.7**
Attrinite	4.7	2.5	3.8	7.2	3.9	4.7	3.8	4.0	**4.3**
Densinite	26.2	20.9	16.6	29.1	29.3	23.5	31.8	33.7	**26.4**
**Gelohuminite**	**7.2**	**3.8**	**6.8**	**9.9**	**9.8**	**8.2**	**5.9**	**6.3**	**7.2**
Corpohuminite	7.2	3.8	6.8	9.9	9.8	8.2	5.9	6.3	**7.2**
**Liptinite (L)**	**13.6**	**14.7**	**16.2**	**17.4**	**15.2**	**12.6**	**9.7**	**10.0**	**13.7**
Sporinite	3.1	4.2	2.6	2.7	3.5	2.4	2.9	2.0	**2.9**
Cutinite	2.3	2.1	1.9	2.1	2.7	2.7	1.3	2.0	**2.1**
Suberinite	0.0	0.0	0.0	0.0	0.0	2.0	0.0	0.0	**0.3**
Resinite	4.9	4.2	7.5	9.9	4.7	3.5	3.8	3.6	**5.3**
Liptodetrinite	3.3	4.2	4.2	2.7	4.3	2.0	1.7	2.4	**3.1**
**Inertinite (I)**	**13.9**	**5.9**	**21.1**	**11.7**	**17.6**	**12.2**	**12.1**	**17.1**	**14.0**
Semifusinite	1.8	0.4	1.9	1.4	3.5	1.2	1.7	2.8	**1.8**
Funginite	8.9	3.8	14.3	8.2	9.4	9.0	7.5	11.5	**9.1**
Inertodetrinite	3.2	1.7	4.9	2.1	4.7	2.0	2.9	2.8	**3.0**
**Mineral Matter (M)**	**12.3**	**21.2**	**8.3**	**8.2**	**3.1**	**17.2**	**17.8**	**10.6**	**12.3**
Others	9.0	14.1	7.5	7.9	2.3	11.8	10.3	8.2	**8.9**
Pyrite	3.3	7.1	0.8	0.3	0.8	5.4	7.5	2.4	**3.5**
Fluorescing H	17.7	15.9	17.0	16.8	18.4	14.9	17.6	19.0	**17.1**
Non-fluorescing (H')	42.5	42.3	37.4	45.9	45.7	43.1	42.8	43.3	**42.9**
Total Fluorescing (H+L)	31.3	30.6	33.2	34.2	33.6	27.5	27.3	29.0	**30.8**
Non-fluorescing (H'+I+M)	68.7	69.4	66.8	65.8	66.4	72.5	72.7	71.0	**69.2**
H (mmf)	68.6	73.9	59.3	68.3	66.2	70.0	73.5	69.7	**68.7**
L (mmf)	15.5	18.7	17.7	19.0	15.7	15.2	11.8	11.2	**15.6**
I (mmf)	15.8	7.5	23.0	12.7	18.2	14.7	14.7	19.1	**15.7**
Rank (R_r%)	0.29	0.28	0.30	0.27	0.27	0.25	0.26	0.27	**0.27**
Standard Deviation	0.02	0.03	0.03	0.04	0.04	0.02	0.03	0.03	**0.03**
Tissue Preservation Index (TPI)	0.86	1.39	1.34	0.69	0.82	0.99	0.64	0.61	**0.92**
Gelification Index (GI)	2.82	6.63	1.94	2.80	2.73	2.79	3.22	2.32	**3.16**
Vegetation Index (VI)	0.91	1.43	1.39	0.79	0.84	1.04	0.71	0.67	**0.97**
Ground Water Index (GWI)	1.71	1.37	1.02	1.99	1.69	1.86	2.44	2.27	**1.79**

Table S4: Values of different recorded macerals and mineral matter (in vol. %), rank (in %) and the various calculated petrographic indices [S3] in the studied samples.

GI = Ulminite + Densinite + Corpohuminite + Gelinite / Textinite + Attrinite + Inertinite

TPI=Textinite + Ulminite + Corpohuminite + Fusinite / Attrinite + Densinite + Gelinite + Inertodetrinite

GWI = Densinite + Corpohuminite + Gelinite + Mineral matter / Textinite + Ulminite + Attrinite

VI=Textinite + Ulminite + Sporinite + Cutinite + Suberinite + Resinite + Fusinite + Semifusinite / Attrinite + Densinite + Alginite + Bituminite + Liptodetrinite + Inertodetrinite

Sample No.	Lithology	CPI	CPI-1	P_{wax}	P_{aq}	OEP 1	OEP 2
U-2	Lignite	0.82	0.82	0.58	0.54	0.60	1.10
U-5	Lignite	1.18	1.18	0.67	0.57	0.64	1.10
U-7	Lignite	1.26	1.26	0.60	0.57	0.81	1.41
U-8	Dark Grey Shale	1.18	1.18	0.75	0.31	0.38	1.20

Table S5: Values of various biomarker parameters of the studied samples.

Serial number	Compound name	m/z
1	Unknown 1	191
2	Methyl Ionene	173
3	Terahydrocadalene	187
4	Possibly C16 cycloalkane	55
5	Calamenene	159
6	Cadalene	183
7	Isocadalene	183
8	Phenanthrene	178
9	Possibly C18 cycloalkane	55
10	16,17-Bisnordehydroabietane	227
11	O1	145
12	Bisnorsimonellite	209
13	Tetrahydroreteme	238
14	Possibly C20 cycloalkane	55
15	Possibly C22 cycloalkane	55
16	Unknown 2	199
17	Des-A-8,14,-seconoroleana5,7,9,13,15,17-hexaene	187
18	3,4,7-Trimethyl-tetrahydrochrysene	259
19	Unknown 3	169
20	Triaromatic C-ring cleaved hydrocarbon	169
21	Dinor-oleana(ursa)-1,3,5 (10) triene	145
22	Perylene	252
23	C- ring cleaved triterpenoids	169
24	Isomer of Dinor-oleana(ursa)-1,3,5 (10) triene	145
25	Isomer of C- ring cleaved triterpenoids	169
26	Dinor-oleana(ursa)-1,3,5(10),12-tetraene	145
27	Isomer of 24,25-dinoroleana(ursa)-1,3,5(10), 12-tetraene	145
28	Tetramethyl diaromatic terpenoid	195
29	Trinor isomer 0f 19	221
30	Lanosta(eupha)pentaene	195
31	Tetranor-oleana-1,3,5(10),6,8,11,13 heptaene	342
32	Isomer of 24,25-dinoroleana(ursa)-1,3,5(10), 12-tetraene	145
33	Lanosta(eupha)hexaene	195
34	C32 Benzohopane	191
35	C33 Benzohopane	191

Table S6: List of compounds identified from the aromatic fractions of the representative sample from Umarsar lignite mines (numbers correspond to the figure S8)

Serial number	Compound name	m/z
a	des-A-oleana-9,13,(18)-diene	326
b	des-A-oleana-5(10),13,18-diene	95
c	des-A-urs-13,(18)-ene	313
d	Olean-2,18-diene	189
e	Trisnorhopene	191
f	A-neoursa-3(5),12-diene	365
g	βTm	149
h	30-Norneohop-13(18)-ene	191
i	Hopene	191
j	Olean-12-ene	218
k	Olean-18-ene	204
l	Olean-2,12-diene	218
m	Hop-17(21)-ene	367
n	Isomer of Olean-12-ene	218
o	Neo-hop-13(18)-ene	191
p	ββ-30-Norhopane	177
q	αβ-Homohopane(22S)	191
r	αβ-Homohopane(22R)	191
s	ββ-homohopane	205
t	ββ-bishomohopane	219

Table S7: List of compounds identified from the saturated fractions of the representative sample from the Umarsar Lignite Mine (numbers correspond to the Figure S9)

Supplemental References

S1 Diessel, C.F., and Diessel, C.F. (1992). Coal facies and depositional environment. Coal-bearing depositional systems, pp.161-264.

S2 Calder, J.H., Gibling, M.R., and Mukhopadhyay, P.K. (1991). Peat formation in a Westphalian B piedmont setting, cumberland basin, Nova Scotia: Implications for the maceral-based interpretation of rheotrophic and raised paleomires. Contribution series No. 91-002.

S3 Kalaitzidis, S., Bouzinos, A., Papazisimou, S., and Christanis, K. (2004). A short-term establishment of forest fen habitat during Pliocene lignite formation in the Ptolemais Basin, NW Macedonia, Greece. International J. Coal Geology, *57*, 243-263.
